# Kiwifruit *Monodehydroascorbate Reductase 3* Gene Negatively Regulates the Accumulation of Ascorbic Acid in Fruit of Transgenic Tomato Plants

**DOI:** 10.3390/ijms242417182

**Published:** 2023-12-06

**Authors:** Dongfeng Jia, Huan Gao, Yanqun He, Guanglian Liao, Liting Lin, Chunhui Huang, Xiaobiao Xu

**Affiliations:** 1 College of Agronomy, Jiangxi Agricultural University, Nanchang 330045, China; dongfengjia@163.com (D.J.); gh11180128@163.com (H.G.); yqhe0312@163.com (Y.H.); liaoguanglian@163.com (G.L.); linliting1998@163.com (L.L.); 2 Institute of Kiwifruit, Jiangxi Agricultural University, Nanchang 330045, China

**Keywords:** ascorbic acid, fruit quality, gene function, kiwifruit, *MDHAR* gene

## Abstract

Ascorbic acid is a potent antioxidant and a crucial nutrient for plants and animals. The accumulation of ascorbic acid in plants is controlled by its biosynthesis, recycling, and degradation. Monodehydroascorbate reductase is deeply involved in the ascorbic acid cycle; however, the mechanism of *monodehydroascorbate reductase* genes in regulating kiwifruit ascorbic acid accumulation remains unclear. Here, we identified seven *monodehydroascorbate reductase* genes in the genome of kiwifruit (*Actinidia eriantha*) and they were designated as *AeMDHAR1* to *AeMDHAR7*, following their genome identifiers. We found that the relative expression level of *AeMDHAR3* in fruit continued to decline during development. The over-expression of kiwifruit *AeMDHAR3* in tomato plants improved monodehydroascorbate reductase activity, and, unexpectedly, ascorbic acid content decreased significantly in the fruit of the transgenic tomato lines. Ascorbate peroxidase activity also increased significantly in the transgenic lines. In addition, a total of 1781 differentially expressed genes were identified via transcriptomic analysis. Three kinds of ontologies were identified, and 106 KEGG pathways were significantly enriched for these differently expressed genes. Expression verification via quantitative real-time PCR analysis confirmed the reliability of the RNA-seq data. Furthermore, *APX3*, belonging to the ascorbate and aldarate metabolism pathway, was identified as a key candidate gene that may be primarily responsible for the decrease in ascorbic acid concentration in transgenic tomato fruits. The present study provides novel evidence to support the feedback regulation of ascorbic acid accumulation in the fruit of kiwifruit.

## 1. Introduction

Ascorbic acid (AsA), also known as vitamin C, is one of the universal antioxidants, and it participates in multiple biological processes in plants [1,2]. Ascorbic acid is also essential for human health, and our bodies have to acquire it mainly from fruits or vegetables because we lack the capacity to synthesize it [3]. Hence, fruit AsA concentration is an important indicator of fruit quality. In recent years, kiwifruit has been highly recognized by consumers due to its high AsA content and unique flavor [4]. However, the mechanism of AsA accumulation in kiwifruit and other horticultural plants is quite complex, and it is closely related to the pathways of biosynthesis, degradation, and recycling [5,6].

It is generally believed that there are four pathways involved in AsA synthesis, namely, the L-galactose pathway, the L-gulose pathway, the galacturonate pathway, and the myo-inositol pathway [7,8,9]. In addition, the accumulation of AsA is also affected by the pathways of recycling and degradation [10,11,12]. Many studies on AsA synthesis in kiwifruit have been carried out [13,14,15,16]; however, studies on the functional identification of key genes related to AsA recycling or degradation are limited.

In some horticultural crops, AsA concentration has been reported to be regulated by the recycling and degradation pathways. For example, in citrus species, in addition to the L-galactose pathway, the recycling pathway also plays important roles in determining AsA content, as high mRNA levels of the *MDHAR3* gene were observed in the fruit of an orange cultivar that accumulates high AsA concentrations [17]. In tomatoes, the expressions of two *MDHAR* genes, *SlMDHAR1* and *SlMDHAR2*, were closely correlated with AsA concentration during fruit ripening, and the authors of the study believed that enhanced AsA recycling activity was responsible for AsA accumulation in the late stages of fruit ripening [18]. On the contrary, over-expression of another tomato *MDHAR* gene in transgenic lines drastically reduced AsA content in mature green tomato fruits [12]. In the fruit of kiwifruit, AsA content is significantly reduced after shading by bagging, which is consistent with the decreased expression of an *MDHAR* gene that is related to AsA metabolism [15]. Therefore, the accumulation level of fruit AsA is closely related to the AsA recycling pathway, and *MDHAR* genes undoubtedly play crucial roles in affecting AsA accumulation in this pathway in fruit crops.

*Actinidia eriantha* is a unique resource in *Actinidia* that is famous for its high AsA concentration and the rich flavor of its fruit [4,19]. *Actinidia eriantha* is an ideal kiwifruit material to explore the mechanism of AsA accumulation in fruit, and the fruit of *A. eriantha* is easy-peel, making it greatly different from the fruits of other kiwifruit species [19]. Although MDHAR is a crucial enzyme in the AsA recycling pathway, the function of *MDHAR* genes in AsA recycling and accumulation has not been clarified in kiwifruit. The purpose of this study was to identify the biological function of key *MDHAR* genes regulating AsA accumulation and to explore the possible routes by which AsA is tightly regulated in the AsA-rich (8.77 mg g^−1^ fresh weight) kiwifruit cultivar, *A. eriantha* ‘Ganmi 6’ [4,14]. This work is helpful in furthering understanding of the effect of the recycling pathway on AsA accumulation in kiwifruit and other fruit crops.

## 2. Results

### 2.1. Kiwifruit MDHAR Family Members and Their Sequence Features

A total of seven *MDHAR* genes were identified in the genome of *A. eriantha* ‘White’, which were named *AeMDHAR1* to *AeMDHAR7*, following their chromosomal locations (Table 1). Each protein sequence of AeMDHARs contained a conserved Pyr_Redox_2 domain (accession number: Pfam07992) (Table S1 from the Supplementary Files). Local BLAST analysis and sequence alignment indicated that these kiwifruit MDHAR proteins share high similarity with their corresponding homologous proteins from Arabidopsis (Table S2 from the Supplementary Files).

As shown in Table 1, these seven kiwifruit *MDHAR* genes are distributed on six chromosomes unevenly. Their CDS lengths and amino acid numbers are very similar, with average values of 1435 bp and 477 amino acid residues, respectively. The molecular weights for these MDHAR proteins were also very close. However, the isoelectric points of different AeMDHAR members were greatly different, likely indicating the multiple functions of different kiwifruit MDHARs.

### 2.2. Phylogenetic Relationships of MDHAR Proteins for Kiwifruit and Other Species

**Figure 1:**
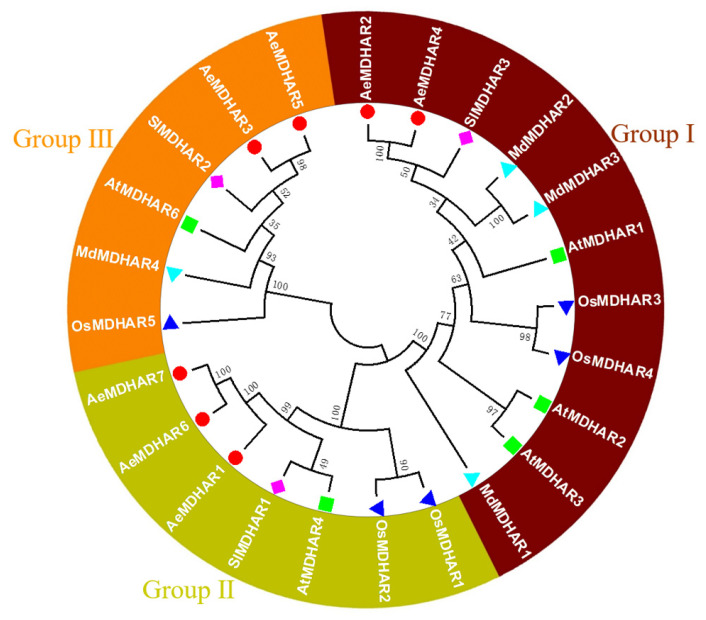
Phylogenetic tree of MDHAR proteins in kiwifruit (*Actinidia eriantha*), Arabidopsis (*Arabidopsis thaliana*), rice (*Oryza sativa*), tomato (*Solanum lycopersicum*), and apple (*Malus domestica*). The different groups are indicated by different colors.

As shown in Figure 1, the 24 MDHAR proteins from five different species were clustered into three groups. For simplicity, these groups were designated Group Ⅰ, Group Ⅱ, and Group Ⅲ. In Group Ⅰ and Group Ⅲ, there were two kiwifruit MDHAR proteins, that is, AeMDHAR2 and AeMDHAR4 in Group Ⅰ, and AeMDHAR3 and AeMDHAR5 in Group Ⅲ. Group Ⅱ included three kiwifruit MDHARs, and they were AeMDHAR1, AeMDHAR6, and AeMDHAR7. Generally, the kiwifruit MDHAR proteins shared close phylogenetic relationships with the corresponding tomato MDHAR proteins. Interestingly, the MDHAR members of apple were only clustered in Group Ⅰ and Group Ⅲ, without any members in Group Ⅱ. In contrast, for the other three species, their MDHAR members were clustered in all three groups (Figure 1).

### 2.3. Structures of AeMDHAR Genes and the Conserved Motifs of AeMDHAR Proteins

**Figure 2:**
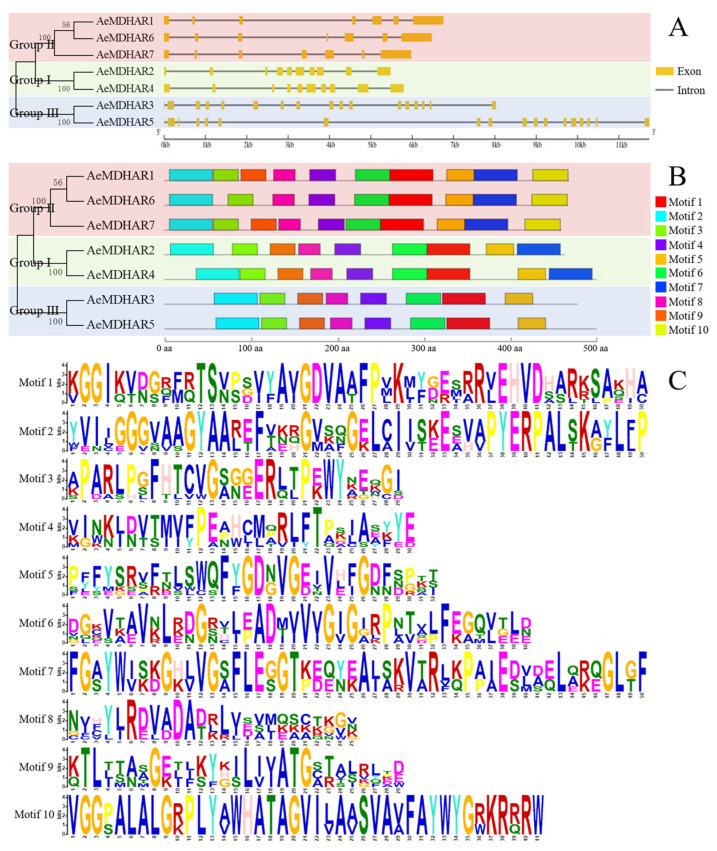
Structures of *AeMDHAR* genes and conserved motifs of AeMDHAR proteins. (**A**). Length and genomic structure of *AeMDHAR* genes. (**B**). Conserved motifs and their distributions of AeMDHAR proteins. (**C**). Amino acid composition of the motifs identified in AeMDHAR proteins. A: alanine. C: cysteine. D: aspartic acid. E: glutamic acid. F: phenylalanine. G: glycine. H: histidine. I: isoleucine. K: lysine. M: methionine. N: asparagine. P: proline. Q: glutamine. R: arginine. S: serine. T: threonine. V: valine. W: tryptophan. Y: tyrosine.

As presented in Figure 2A, the gene structures in different MDHAR groups were obviously different. The numbers of exons and introns for genes within different groups were greatly different. For Group Ⅱ, each *AeMDHAR* gene contained 7 exons and 6 introns; for Group Ⅰ, both of the two members contained 10 exons and 9 introns; for Group Ⅲ, *AeMDHAR3* included 17 exons and 16 intron, while *AeMDHAR5* contained the most numbers of exons and introns, that is, 18 exons and 17 introns (Figure 2A).

A total of 10 conserved motifs were identified in the seven AeMDHAR proteins, which were named motif 1 to motif 10 (Figure 2B). Among them, seven motifs were found to exist in all of the seven AeMDHAR proteins, which were motif 1, motif 2, motif 3, motif 4, motif 5, motif 6, and motif 8. The motif type and distribution in proteins within a same group were very similar. For example, in Group Ⅰ, both AeMDHAR2 and AeMDHAR4 contained nine conserved motifs, and they were distributed in the same order. Similar situations also occurred for AeMDHAR3 and AeMDHAR5 in Group Ⅲ. For the three AeMDHARs in Group Ⅱ, two proteins, AeMDHAR1 and AeMDHAR7, contained all 10 conserved motifs, while AeMDHAR6 lacked motif 9. In addition, motif 10 was found to be the specific motif that only existed in the proteins of Group Ⅱ (Figure 2B). For those 10 motifs, there were differences in amino acid number and amino acid composition. For example, motif 1, motif 2, and motif 7 each contained 50 amino acid residues, while motif 8 only included 25 amino acid residues (Figure 2C, Table S3 from the Supplementary Files). For most motifs, the amino acid type at a certain site was different; however, for motif 10, only one amino acid residue was found at more than 80% of sites (Figure 2C). In short, we identified 10 conserved motifs in the sequences of the seven kiwifruit MDHAR proteins, and those motif sequences with the most likely amino acid residues at each site are shown in Table S3 in the Supplementary Files.

### 2.4. Synteny Relationships of MDHAR Genes within Kiwifruit Genome and between Genomes of Kiwifruit and Arabidopsis

**Figure 3:**
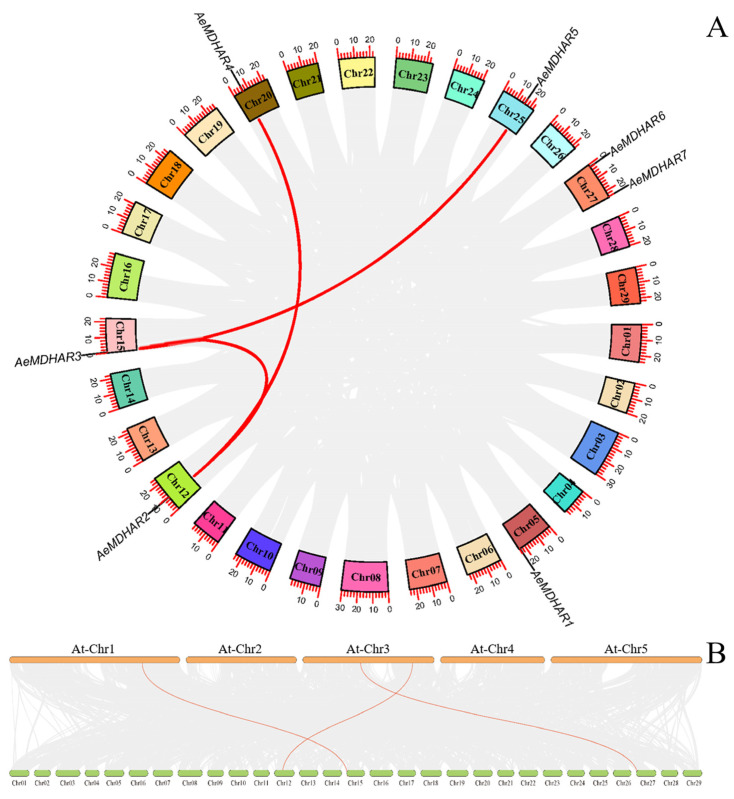
Synteny relationships of *MDHAR* genes within kiwifruit (*Actinidia eriantha*) genome (**A**) and between the genomes of kiwifruit and Arabidopsis (*Arabidopsis thaliana*) (**B**). The gene pairs with synteny relationship are linked by red or pink lines.

Within the genome of *A. eriantha* ‘White’, three *MDHAR* gene pairs with synteny relationships were identified, which included *AeMDHAR2*/*AeMDHAR3*, *AeMDHAR2*/*AeMDHAR4*, and *AeMDHAR3*/*AeMDHAR5* (Figure 3A).

Additionally, for *MDHAR* gene family members between kiwifruit and Arabidopsis, three gene pairs across the two species exhibited a synteny relationship, which were *AeMDHAR2*/*AtMDHAR4*, *AeMDHAR3*/*AtMDHAR1*, and *AeMDHAR6*/*AtMDHAR3* (Figure 3B).

### 2.5. Relative Expressions of Kiwifruit MDHAR Genes in Different Tissues and in Fruits with Different Developmental Stages

**Figure 4:**
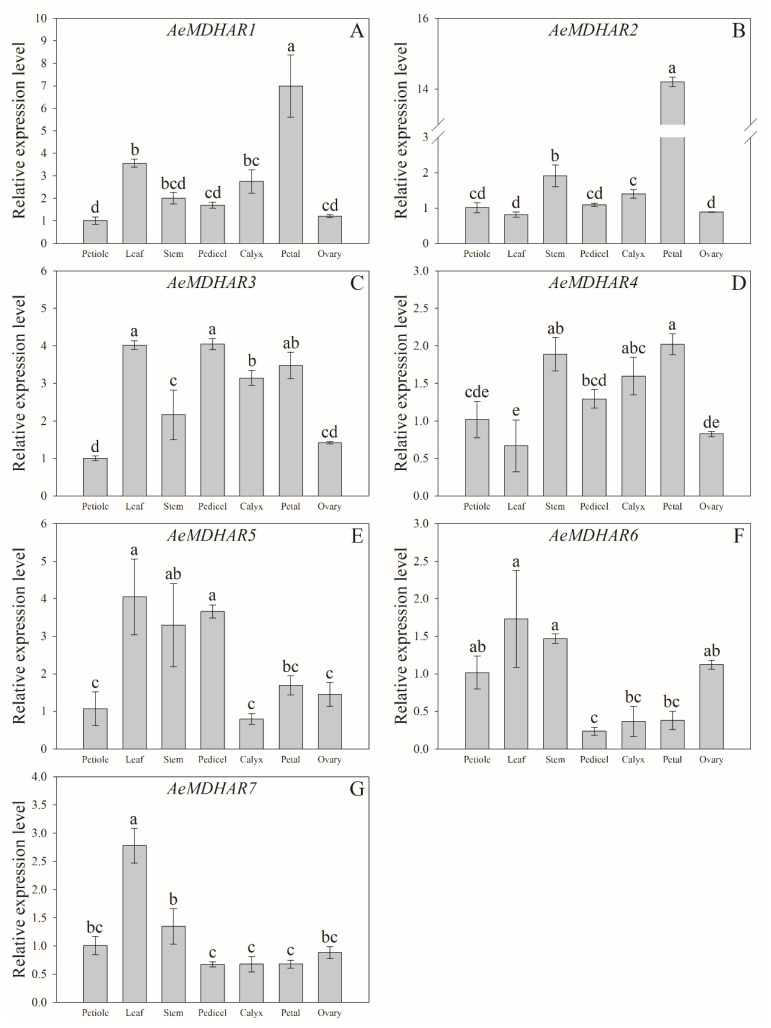
Relative expression levels of kiwifruit *MDHAR* genes in different tissues of *Actinidia eriantha* ‘Ganmi 6’. (**A**). The relative expression level of *AeMDHAR1*. (**B**). The relative expression level of *AeMDHAR2*. (**C**). The relative expression level of *AeMDHAR3*. (**D**). The relative expression level of *AeMDHAR4*. (**E**). The relative expression level of *AeMDHAR5*. (**F**). The relative expression level of *AeMDHAR6*. (**G**). The relative expression level of *AeMDHAR7*. The petiole samples were used as control to calculate the relative expression levels among different tissues. Different letters above the bars indicate the significant differences among the data at a level of *p* < 0.05 according to Tukey’s multiple comparison test.

The expression analysis suggests that the seven *AeMDHAR* genes all expressed in all the samples of *A. eriantha* ‘Ganmi 6’ (Figure 4). For convenience, the petiole sample was used as the control to compare the relative gene expression levels among different tissues. For *AeMDHAR1* and *AeMDHAR2*, the relative expressions in petals were significantly higher than those in other kiwifruit tissues (Figure 4A,B). For *AeMDHAR3* and *AeMDHAR4*, the differences in expression levels in different tissues were very small (Figure 4C,D). The relative expression levels of *AeMDHAR5* in the leaf, stem, and pedicel were significantly higher than those in the petiole and calyx (Figure 4E). For *AeMDHAR6*, the expressions in the leaf and stem were very high, while they were significantly lower in pedicel, calyx, petal, and fruit (Figure 4F). The relative expression level of *AeMDHAR7* in leaf was significantly higher than that in all the other tissues (Figure 4G).

**Figure 5:**
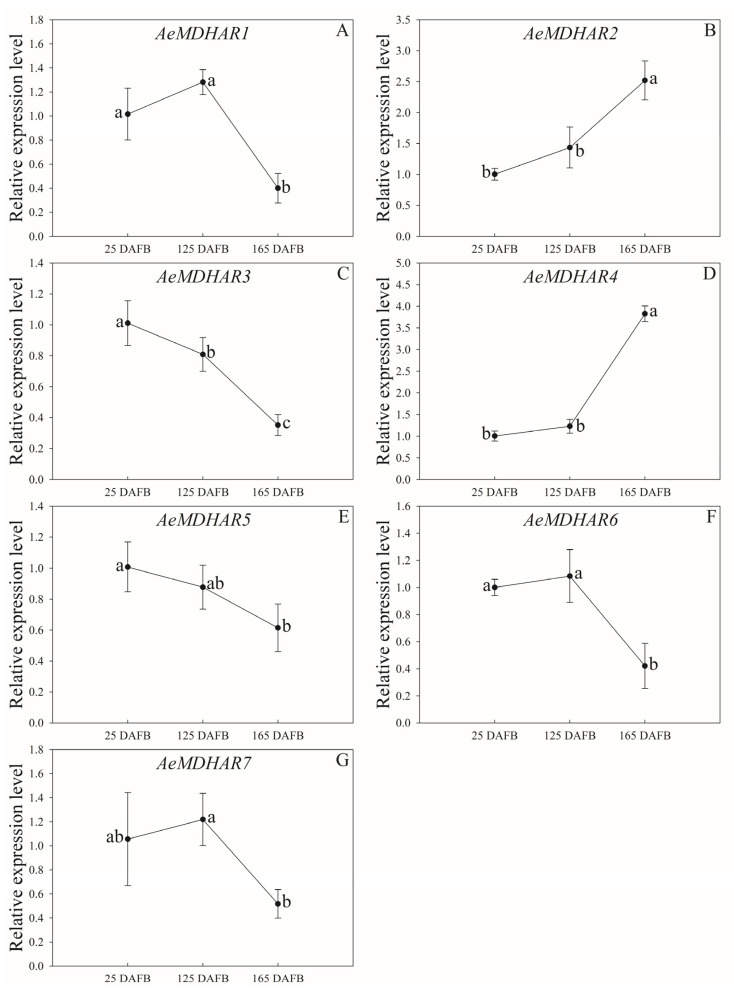
Relative expression levels of kiwifruit *MDHAR* genes in *Actinidia eriantha* ‘Ganmi 6’ fruits with different developmental stages. (**A**). The relative expression level of *AeMDHAR1* in developmental fruits. (**B**). The relative expression level of *AeMDHAR2* in developmental fruits. (**C**). The relative expression level of *AeMDHAR3* in developmental fruits. (**D**). The relative expression level of *AeMDHAR4* in developmental fruits. (**E**). The relative expression level of *AeMDHAR5* in developmental fruits. (**F**). The relative expression level of *AeMDHAR6* in developmental fruits. (**G**). The relative expression level of *AeMDHAR7* in developmental fruits. The fruit samples collected on 25th day after full bloom (DAFBs) was used as control to calculate the relative expression levels. Different letters beside the bars indicate the significant differences among the data at a level of *p* < 0.05 according to Tukey’s multiple comparison test.

As shown in Figure 5, during the developmental process from the 25th to 165th day after full bloom (DAFB), the relative expression levels of the seven *AeMDHAR* genes roughly exhibited three patterns: the stable-decreasing type, the stable-increasing type, and the decreasing type. For *AeMDHAR1*, *AeMDHAR6*, and *AeMDHAR7*, their relative expression levels did not change significantly from 25 to 125 DAFB; however, during the following period from 125 to 165 DAFBs, the expressions for these three genes significantly decreased (Figure 5A,F,G); thus, the expression patterns of these genes belonged to the stable-decreasing type. In addition, the expression patterns of two *AeMDHAR* genes, *AeMDHAR2* and *AeMDHAR4*, tended to be the stable-increasing type. That is, there were no significant differences for the relative expression levels of *AeMDHAR6* and *AeMDHAR7* from 25 to 125 DAFBs; on the contrary, during the developmental process from 125 to 165 DAFBs, the expression levels of these two genes were significantly higher comparing with that during the previous period (Figure 5B,D). In addition, the expression patterns of *AeMDHAR3* and *AeMDHAR5* seemed to be the decreasing type. During the developmental process from 25 to 165 DAFBs, the relative expression levels continued to fall (Figure 5C,E). In particular, for *AeMDHAR3*, the relative expression level at 125 DAFBs was 80.05% compared with that at 25 DAFBs, and at 165 DAFBs, it was 34.81% and 43.49% when compared with that at 25 and 125 DAFBs, respectively, significantly lower than that at the previous two stages (Figure 5C).

### 2.6. Molecular Features, Phylogenetic Relationships, and Sequence Similarities of AeMDHAR3 and Homologous Proteins from Other Species

**Figure 6:**
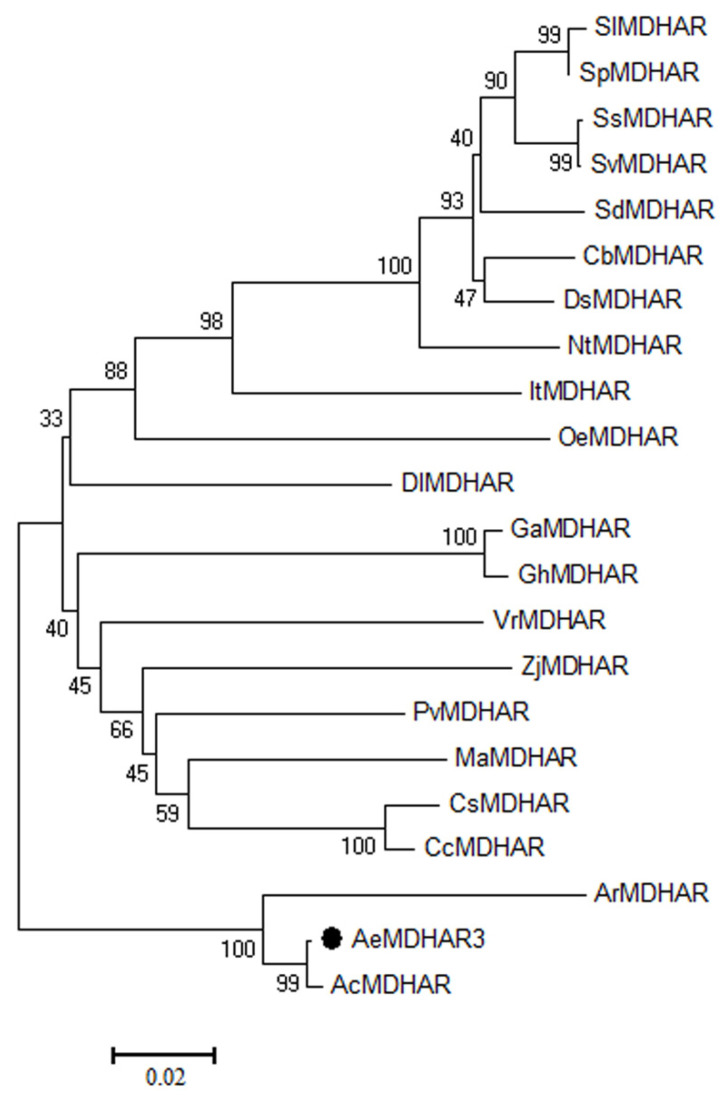
Phylogenetic tree of AeMDHAR3 (marked with solid circle) and its homologous proteins from other species.

**Figure 7:**
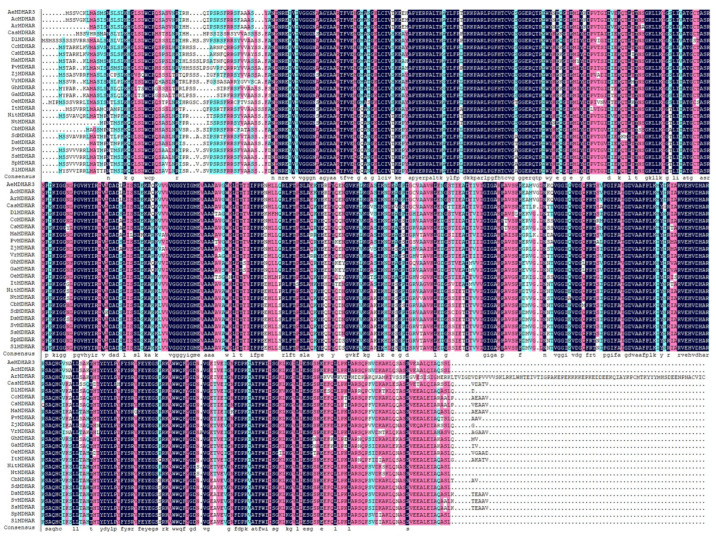
Similarity of AeMDHAR3 and its homologous proteins from other species. These different colors show the similarity degree of the amino acids among the MDHAR proteins.

*Actinidia eriantha monodehydroascorbate reductase 3* (*AeMDHAR3*) was cloned from a fruit sample of *A. eriantha* ‘Ganmi 6’. The CDS of this gene included 1476 nucleotides, encoding a protein containing 488 amino acid residues (Table S4 from the Supplementary Files). A sequence alignment indicated that the similarity between the cloned CDS and the predicated CDS of this gene obtained from the kiwifruit genome was 96.20% (Figure S1A from the Supplementary Files), and the similarity between the protein sequences was 91.21% (Figure S1B from the Supplementary Files). The phylogenetic analysis and sequence alignment showed that the protein sequence of AeMDHAR3 shared high similarity with its homologous proteins of two other kiwifruit species, i.e., *A. chinensis* and *A. rufa* (Figure 6 and Figure 7). Specifically, AcMDHAR (PSS09385.1) of *A. chinensis* was the most similar homologous protein with AeMDHAR3, and their similarity was 99.39%. Meanwhile, the similarity of AeMDHAR3 and the homologous ArMDHAR (GFZ01457.1) from *A. rufa* was 79.36%. These MDHAR proteins from three different *Actinidia* species clustered into a single branch in the phylogenetic tree (Figure 6).

### 2.7. Expression of AeMDHAR3 and Activity of MDHAR in Transgenic Tomato Lines

**Figure 8:**
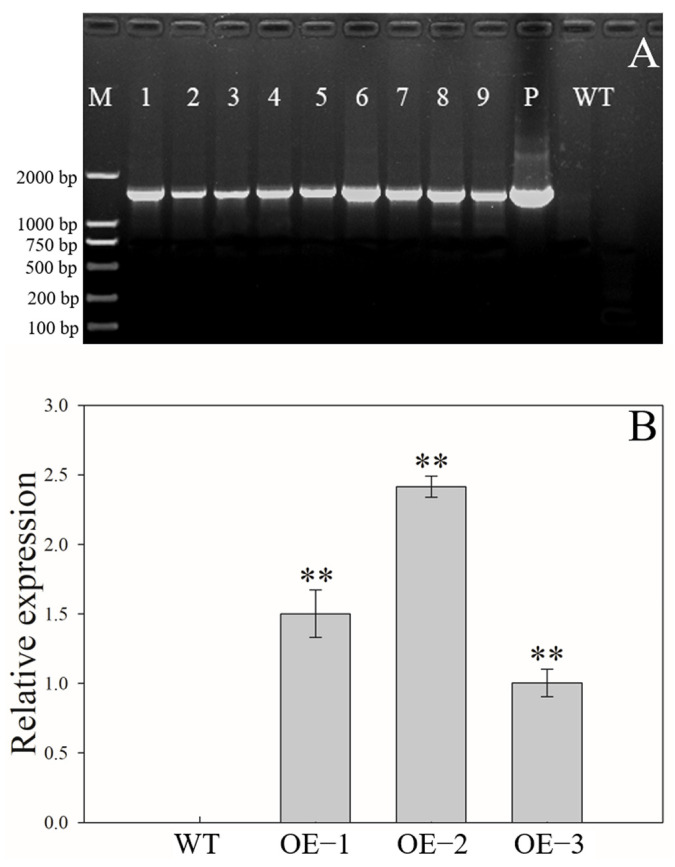
Test results of transgenic tomato lines overexpressing *AeMDHAR3* (**A**) and the relative expressions of *AeMDHAR3* in three over-expressed transgenic tomato lines (**B**). bp: base pair. M: DNA marker. P: positive control. WT: wild-type. OE: over-expressed. “**” above the bars represents the significant difference between the OE and the WT tomato plants according to independent-samples T test method at *p* < 0.01 level.

A total of nine transgenic tomato lines over-expressing *AeMDHAR3* were obtained (Figure 8A). Three T_1_ generation transgenic tomato lines (OE−1, OE−2, and OE−3) were used for further analysis. The expression of *AeMDHAR3* was found in the leaves of all the three OE lines, while no expression was detected in the leaves of the WT tomato plants (Figure 8B). Among these transgenic lines, the highest relative expression level of *AeMDHAR3* was found in OE−2, followed by OE−1 and OE−3 (Figure 8B).

**Figure 9:**
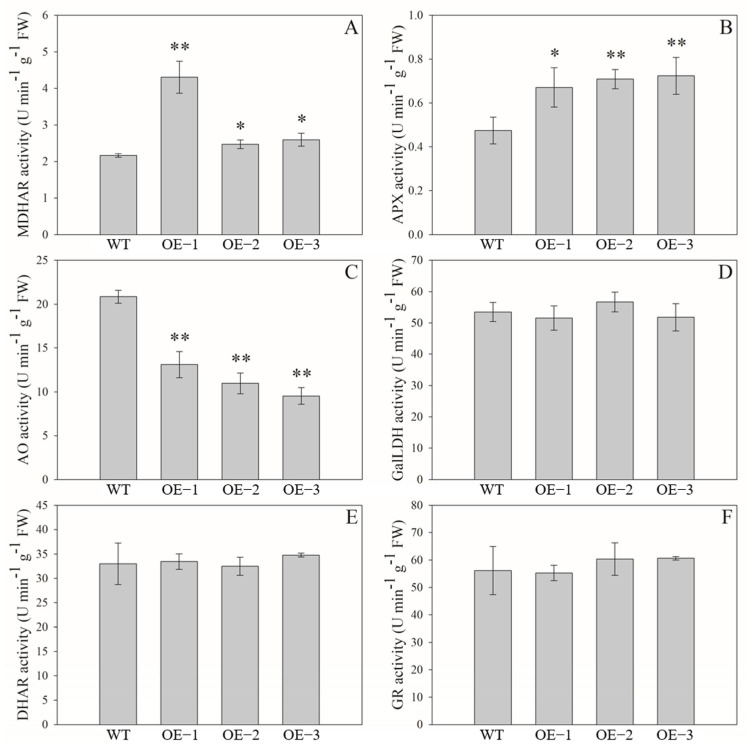
Activities of enzymes related to AsA accumulation in fruits of wild-type tomato plants (WT) and three transgenic tomato lines over-expressing *AeMDHAR3* (OE). (**A**). The activity of MDHAR in OE and WT tomato plants. (**B**). The activity of APX in OE and WT tomato plants. (**C**). The activity of AO in OE and WT tomato plants. (**D**). The activity of GalLDH in OE and WT tomato plants. (**E**). The activity of DHAR in OE and WT tomato plants. (**F**). The activity of GR in OE and WT tomato plants. “**” and “*” above the bars represent the significant difference between the OE and the WT tomato plants according to independent-samples T test method at *p* < 0.01 level and *p* < 0.05 level, respectively.

As shown in Figure 9, MDHAR activity in fruits of the OE lines was significantly higher than that of the WT plants; in particular, in the OE−1 line, the value was 4.3 U min^−1^ g^−1^ fresh weight (FW), almost two times higher than that in WT plants (Figure 9A). The activity of APX, an important enzyme catalyzing AsA into MDHA, was significantly higher in these OE lines, and their values increased about 47% when comparing with that of WT plants (Figure 9B). By contrast, for those transgenic tomato lines, the values of AO activity were significantly lower than that of WT plants (Figure 9C). In addition, for the three enzymes of GalLDH, DHAR, and GR, their activities in transgenic tomato fruits were not significantly affected by the over-expression of *AeMDHAR3* (Figure 9D–F).

### 2.8. Contents of AsA and DHA in Transgenic Tomato Lines

**Figure 10:**
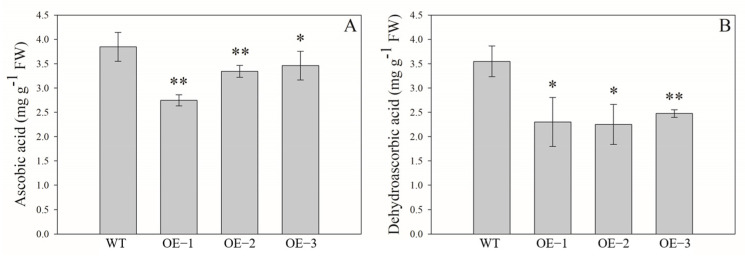
The contents of AsA (**A**) and DHA (**B**) in fruits of wild-type tomato plants (WT) and the three transgenic tomato lines over-expressing AeMDHAR3 (OE−1, OE−2, and OE−3). “**” and “*” above the bars represent the significant difference between the OE and the WT tomato plants according to independent-samples T test method at *p* < 0.01 level and *p* < 0.05 level, respectively.

The contents of AsA and its oxidation state, DHA, were respectively detected in fruits of these OE lines. Unexpectedly, the AsA content in the fruit of transgenic tomato lines over-expressing *AeMDHAR3* was significantly lower than that in WT plants, and the values of those OE lines were between 71.4% and 89.9% compared with that of the control (Figure 10A). Meanwhile, the DHA content was also significantly lower in tomato fruit of the OE lines when compared with WT, decreased by about 34% (Figure 10B).

### 2.9. Differently Expressed Genes and Their GO Enrichment and KEGG Pathway Enrichment

For all the fruit samples collected from WT (WT−1, WT−2, and WT−3) or OE−1 plants (OE−1−1, OE−1−2, and OE−1−3), 41,434,366 to 91,422,210 clean reads were obtained from RNA-seq analysis (Table 2). For those reads, Q20 and Q30 were more than 96.0% and 91.5%, respectively. In addition, the ratios of GC content were between 42.10% and 42.30% (Table 2). These results suggest that the quality of the sequencing was high enough.

**Figure 11:**
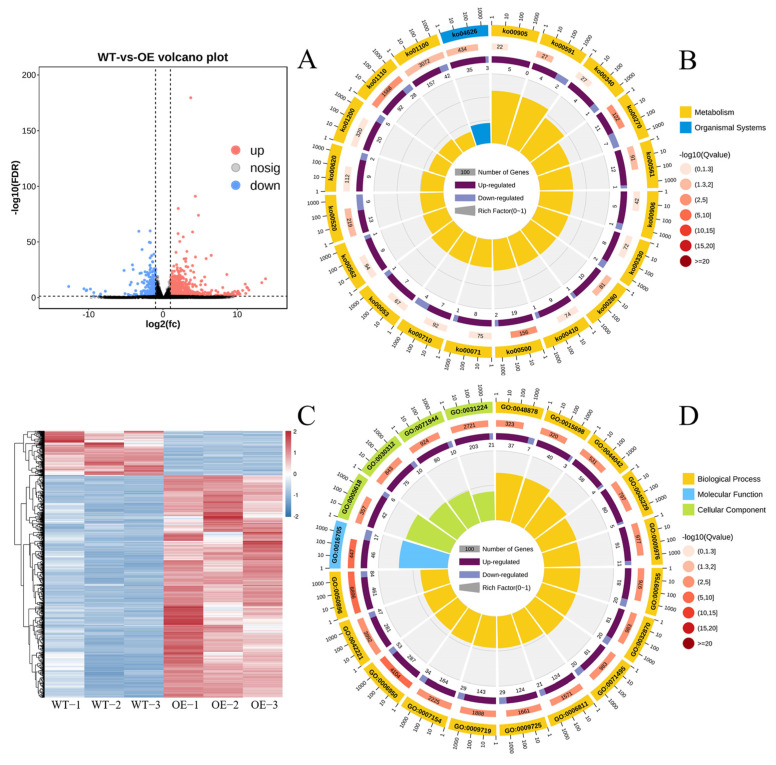
The differently expressed genes (DEGs) and the related gene expression profiles, functional classifications, and pathway annotations. (**A**). Volcano map of DEGs between OE−1 and WT tomato plants. (**B**). Functional classification obtained via GO enrichment analysis for DEGs between OE−1 and WT tomato plants. (**C**). Clustering heat map of DEGs between OE−1 and WT tomato plants. (**D**). Significant pathways identified via KEGG enrichment analysis for DEGs between OE−1 and WT tomato plants.

In addition, a total of 1781 genes were identified as DEGs between OE−1 and WT plants. Among them, 1486 genes (83.44%) were up-regulated genes in the OE−1 line, and 295 genes (16.56%) were down-regulated genes (Figure 11A). The volcano map shows the distribution of those up-regulated and down-regulated DEGs in OE−1 plants (Figure 11A). As shown in Figure 11C, the expression patterns of those DEGs clustered into the same branch were very similar in the replicated samples, suggesting that they may participate in same metabolic or signal transduction pathways.

The GO enrichment analysis indicated that those DEGs between OE−1 and WT plants could be classified into three ontologies: molecular function, cellular component, and biological process. The DEGs within each ontology could be further classified into sub-GO terms. The top 20 significantly enriched GO terms were selected, they included 15 DEGs within the ontology of biological process, 4 DEGs within the ontology of cellular component, and 1 DEG within the ontology of molecular function (Figure 11B). For the biological process ontology, response to stimulus (GO:0050896), response to stress (GO:006950), and response to chemical (GO:0042221) were the three GO terms with the most significant differences. Among the members of response to stimulus term, there were 545 gene members, including 461 up-regulated DEGs and 84 down-regulated DEGs. For the GO term of response to stress, 340 DEGs were identified, and 287 of them were up-regulated and 53 were down-regulated in the OE−1 line. The term of response to chemical contained 328 DEGs, with 281 up-regulated genes and 47 down-regulated genes (Figure 11B). For the cellular component ontology, cell wall (GO:0005618) and intrinsic component of membrane (GO:0031224) were the most significant GO terms. The number of DEGs in the cell wall term was 48, including 42 up-regulated genes and 6 down-regulated genes. The term intrinsic component of membrane contained 224 DEGs, including 203 up-regulated genes and 21 down-regulated genes (Figure 11B). For the ontology of molecular function, the GO term of oxidoreductase activity (GO:0016705) was the most significant term between the plants of WT and OE−1. We identified 63 DEGs within this term; they included 46 up-regulated genes and 17 down-regulated genes (Figure 11B).

The KEGG pathway enrichment analysis suggested that a total of 106 KEGG pathways were significantly enriched for those 1781 DEGs (Table S5 from the Supplementary Files). Two types of pathways were found for the top 20 significantly enriched pathways, that is, metabolism and organismal systems (Figure 11D). Specifically, the pathways with the greatest significant differences were the biosynthesis of secondary metabolites (ko01110) (120 DEGs), cysteine and methionine metabolism (ko00270) (18 DEGS); starch and sucrose metabolism (ko00500) (21 DEGs); metabolic pathways (ko01100) (199 DEGs); glycerolipid metabolism (ko00561) (13 DEGs); plant–pathogen interaction (ko04626) (38 DEGs); linoleic acid metabolism (ko00591) (6 DEGs); amino sugar and nucleotide sugar metabolism (ko00520) (22 DEGs); valine, leucine and isoleucine degradation (ko00280) (11 DEGs); arginine and proline metabolism (ko00330) (10 DEGs); brassinosteroid biosynthesis (ko00905) (5 DEGs); beta-Alanine metabolism (ko00410) (10 DEGs); carbon fixation in photosynthetic organisms (ko00710) (11 DEGs); histidine metabolism (ko00340) (5 DEGs); fatty acid degradation (ko00071) (9 DEGs); carotenoid biosynthesis (ko00906) (6 DEGs); ascorbate and aldarate metabolism (ko00053) (8 DEGs); inositol phosphate metabolism (ko00562) (10 DEGs); pyruvate metabolism (ko00620) (11 DEGs); and carbon metabolism (ko01200) (25 DEGs). Totally, among all the 558 DEGs within the 20 pathways, 444 genes were up-regulated in the OE line (79.6%), and 114 were down-regulated genes (20.4%) (Figure 11D).

### 2.10. Results of Expression Verification of Related Differently Expressed Genes

**Figure 12:**
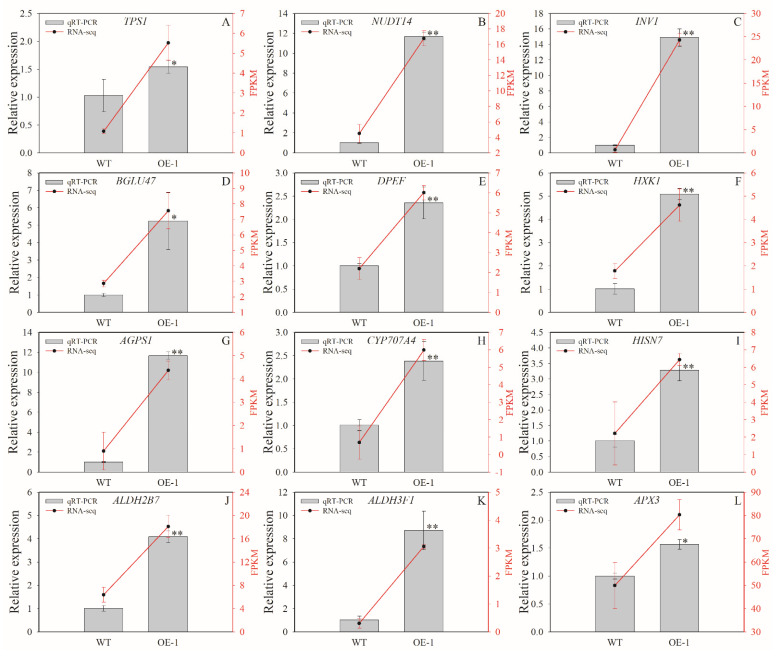
Expressions of differentially expressed genes between the over-expressed transgenic line (OE−1) and the wild-type tomato plants. The subfigures of (**A**–**L**) represent the gene expression data of *TPS1*, *NUDT14*, *INV1*, *BGLU47*, *DPEF*, *HXK1*, *AGPS1*, *CYP707A4*, *HISN7*, *ALDH2B7*, *ALDH3F1*, and *APX3*, respectively, in OE−1 line over-expressing kiwifruit *AeMDHAR3* gene. Gray columns represent the relative expression levels of genes analyzed via qRT-PCR method. Red lines indicate the expression levels of DEGs via FPKM values of transcriptome analysis. “**” and “*” beside the black bars represent the significant difference between the OE−1 and the WT tomato plants according to independent-samples T test method at *p* < 0.01 level and *p* < 0.05 level, respectively.

Comparing with WT, the transcriptome data suggest that all the 12 DEGs exhibited improved expressions in the OE−1 line (Figure 12). The expression verification via qRT-PCR analysis also indicates that the relative expression levels of those 12 genes were significantly higher in the OE−1 line when compared with that in WT plants (Figure 12). Thus, the expression trends are highly consistent with the results of RNA-seq analysis, demonstrating that the RNA-seq data are reliable and suitable for further analysis.

Furthermore, for *NUDT14* and *INV1*, two genes within the KEGG pathway of starch and sucrose metabolism, their FPKM values were 16.78 and 24.29, respectively, in the fruit of the OE−1 line, much higher than that in WT plants (Figure 12B,C); similarly, their relative expression levels in the OE−1 line were both more than 11 times higher than that in the control (Figure 12B,C). In addition, for *APX3* within the KEGG pathway of ascorbate and aldarate metabolism, the FPKM value was very high in fruit samples of the transgenic line (Figure 12L); its relative expression level was also significantly higher in the OE−1 line compared to that in WT plants (Figure 12L). These results possibly imply the important role of the *APX3* gene in regulating AsA metabolism in tomato fruits.

## 3. Discussion

MDHAR is an especially important enzyme for AsA regeneration in the AsA recycling pathway [10]; additionally, MDHAR plays essential functions in cell metabolism and in responses to abiotic stresses [20]. In several horticultural crops, such as spinach [21], pea [22], and tomato [18], *MDHAR* genes have been cloned. However, no systematic study has been performed on *MDHAR* family genes in kiwifruit. In this work, we identified all the *MDHAR* family members in the genome of kiwifruit (*A. eriantha*) for the first time.

The expressions of the seven *AeMDHAR* genes were detected in all those related kiwifruit tissues, as well as in fruits with different developmental stages (Figure 4 and Figure 5). These results indicate that *MDHAR* genes are crucial to organ growth and fruit development in kiwifruit. Similarly, in Arabidopsis, a *MDHAR* gene, *MDAR4*, is critical for the recycling of AsA to maintain the growth of seedlings [23]. Additionally, according to our previous reports, fruit AsA content was generally reduced with fruit growth and development in *A. eriantha* [4,24]; combining this with the present study, where we found that the expression trend of *AeMDHAR3* in fruit was consistent with the changes in AsA content, we thus believe that *AeMDHAR3* likely plays a crucial role in fruit AsA regeneration.

As the transformation of genes into kiwifruit is difficult and time-consuming, *AeMDHAR3* was cloned from *A. eriantha* ‘Ganmi 6’ and was transformed into tomato plants. High expressions of *AeMDHAR3* were detected in those OE tomato lines (Figure 8). As expected, MDHAR activity was significantly higher in fruits of OE lines than in non-transformed WT plants; particularly, the value of MDHAR activity in OE−1 increased by 99.0% when compared with the control (Figure 9A). Unexpectedly, in fruits of those transgenic tomato lines, the AsA contents were all significantly lower than that in WT, decreasing by 10.1% to 28.6% (Figure 10A). These results are not consistent with many previous reports. For example, in tomato (*Lycopersicon esculentum*), overexpression of a *MDHAR* gene, *LeMDAR*, markedly increased MDHAR activity and AsA level [25]. Similarly, ectopic over-expression of an *A. thaliana MDAR* gene (*AtMDAR1*) in tobacco (*Nicotiana tabacum*) leads to 2.1-fold higher MDHAR activity and 2.2-fold higher AsA level in transgenic tobacco plants [26]. On the other hand, there is some evidence that *MDHAR* genes negatively regulate AsA content in plants. For instance, in mature green fruits of transgenic tomato lines over-expressing a *S. lycopersicum MDHAR* gene, the AsA level significantly reduced by 30–50% [12]. In addition, a previous study indicated that the transcript levels of two *MDHAR* genes were negatively correlated with AsA levels during fruit ripening in tomatoes [27]. Additionally, for a cherry tomato cultivar, *S. lycopersicum* ‘West Virginia 106’, AsA levels in leaves decreased apparently in transgenic lines over-expressing the *MDHAR3* gene, whereas in the silenced lines, AsA levels increased significantly in both fruits and leaves [28]. Therefore, the previous scientific literature combined with our research confirms that MDHAR is a key enzyme that strongly affects AsA regeneration in specific plant species. Furthermore, the opposite effects of *MDHAR* genes on AsA accumulation in different plants indicate that *MDHAR* genes function in a species-specific manner.

To further explore the reason why the over-expression of *AeMDHAR3* significantly restricted AsA accumulation in transgenic tomato fruits, the activities of several enzymes related to AsA biosynthesis, degradation, or recycling were determined. Interestingly, the activities of GalLDH, DHAR, and GR were not obviously affected by the promoted expression of *AeMDHAR3*; however, significant changes in the activities of APX and AO were observed (Figure 8). Although these two enzymes are both responsible for the oxidation of AsA in the recycling pathway, their changing patterns were opposite: APX activity increased significantly, whereas AO activity decreased in the OE lines (Figure 9B,C).

In plants, the regulation of AsA concentration is a complex biological process, and the pathways of AsA biosynthesis, degradation, and recycling are all closely related to AsA level [17]. During the process of AsA metabolism, it is firstly transformed into MDHA catalyzed by AO or APX, then MDHA can either be recycled into AsA catalyzed by MDHAR or be disproportionated to AsA and DHA [6]. Moreover, the oxidized AsA state, DHA, can be recycled into the reduced state (AsA) via the ascorbate-glutathione (AsA-GSH) cycle [29]. In the AsA-GSH cycle, if not rapidly reduced to AsA, DHA can also be irreversibly hydrolyzed, causing the loss of the total AsA pool [6]. In the AsA recycling pathway, the change in activity of one enzyme may significantly affect the activities of other enzymes [30]. Similarly, in this study, MDHAR activity was significantly improved in transgenic tomato fruits by the over-expression of *AeMDHAR3*, and a significant increase in APX activity was observed in those OE lines. The promotion of MDHAR activity is helpful for the instantaneous increase of AsA concentration, and the instantaneous increase in AsA concentration probably enhanced the activity of APX, leading to more degradation of AsA in tomato fruit. Thus, APX activity in tomatoes may be partially affected by substrate concentration, that is, AsA concentration. Therefore, MDHAR and APX probably function in a coordinated way to regulate AsA recycling. We speculate that the oxidation rate of AsA catalyzed by APX was higher than its regeneration rate catalyzed by MDHAR in transgenic tomato plants. In addition, for the OE lines, AO activity was significantly lower than that in WT plants, which also proves our inference that APX possibly plays a crucial role in the oxidation of AsA into MDHA.

The KEGG enrichment analysis indicates that many DEGs were enriched in the pathway of starch and sucrose metabolism (Figure 11D), implying that *AeMDHAR3* may be also involved in sugar accumulation. Interestingly, in cherry tomatoes (*S. lycopersium*), the silencing of a *MDHAR* gene results in low accumulations of hexoses and sucrose in a light-dependent manner [31]. More studies should be conducted to explore the possible function of kiwifruit *MDHAR* genes in regulating sugar accumulation. Moreover, eight DEGs were enriched in the pathway of ascorbate and aldarate metabolism (Figure 11D). Among them, the expression of *APX3* was significantly higher in the OE−1 line (Figure 12L), suggesting the important role of *APX3* gene in regulating AsA accumulation in tomatoes. As a result, we further assumed that the improved activity of APX in transgenic tomato plants may be not only attributed to the instantaneous increase in AsA concentration, but also related to the enhanced transcriptional levels of the *APX3* gene.

## 4. Materials and Methods

### 4.1. Plant Materials

Three uniform vines of *A. eriantha* ‘Ganmi 6’ planted at the Kiwifruit Institute of Fengxin County (28°70′ N, 115°38′ E), Jiangxi Province, China were used in this study. Different tissue samples (petiole, leaf, stem, pedicel, calyx, petal, and ovary) and fruit samples from three developmental stages (25, 125, and 165 DAFBs) were collected. After collection, the samples were packaged and moved to our laboratory immediately. For fruit samples, the pulp tissue (outer pericarp) was separated and cut into pieces, frozen in liquid nitrogen, and stored at −80 °C. The other samples were also cut into pieces, frozen in liquid nitrogen, and stored at −80 °C. Three biological replicates were performed for all the samples.

### 4.2. Genome-Wide Identification of MDHAR Protein Family Members in Kiwifruit (Actinidia eriantha)

All the MDHAR protein sequences of Arabidopsis were downloaded from the Arabidopsis Information Resource (https://www.arabidopsis.org/, accessed on 1 May 2022). The AtMDHAR sequences were used as queries to conduct a local BLASTP search using BioEdit software (version 7.0.9, Ibis BioSciences, Carlsbad, CA, USA) against all the predicted protein sequences (downloaded from the Kiwifruit Genome Database, http://kiwifruitgenome.org/, accessed on 1 May 2022) of *A. eriantha* ‘White’ according to a previous method [32]. For the candidate sequences, hidden Markov models and the Pfam database (http://pfam.xfam.org/, accessed on 1 May 2022) were used to identify the Pyr_Redox_2 domain, and those protein sequences without an intact Pyr_Redox_2 domain were excluded. Furthermore, the sequences were carefully examined manually to exclude the ones with any ambiguous domain that belonged to other protein families. The remaining candidate sequences were identified as the members of MDHAR protein family in kiwifruit (*A. eriantha*) and they were used for further analysis.

### 4.3. Phylogenetic Analysis for MDHAR Proteins of Kiwifruit and Other Species

The MDHAR protein sequences of Arabidopsis (*Arabidopsis thaliana*), rice (*Oryza sativa*), tomatoes (*Solanum lycopersicum*), and apples (*Malus domestica*) were downloaded from the UniProt database (https://www.uniprot.org/, accessed on 1 May 2022) or the GDR database (https://www.rosaceae.org/, accessed on 1 May 2022), respectively. They included 5 Arabidopsis MDHARs (AtMDHAR1/AT3G52880.2, AtMDHAR2/AT5G03630.1, AtMDHAR3/AT3G09940.1, AtMDHAR4/AT3G27820.1, AtMDHAR6/AT1G63940.2), 5 rice MDHARs (OsMDHAR1/Os02t0707000-00, OsMDHAR2/Os02t0707100-01, OsMDHAR3/Os09t0567300-01, OsMDHAR4/Os08t0557600-01, OsMDHAR5/Os08t0151800-01), 3 tomato MDHARs (SlMDHAR1/Solyc02g086710.2.1, SlMDHAR2/Solyc08g081530.2.1, SlMDHAR3/Solyc09g009390.2.1), and 4 apple MDHARs (MdMDHAR1/MD11G0190600, MdMDHAR2/MD12G0020500, MdMDHAR3/MD14G0017300, MdMDHAR4/MD14G0096900). Multiple sequence alignment was performed for these MDHAR proteins and kiwifruit MDHAR proteins using the MUSCLE method, and a phylogenetic tree was constructed via the neighbor-joining method with 1000 replicates using MEGA software (version 5.05, Temple University, Philadelphia, PA, USA) [32].

### 4.4. Analyses of Genomic Structure and Sequence Features for MDHAR Genes

Information regarding genomic nucleotide sequences and their corresponding coding sequences (CDSs) of kiwifruit *MDHAR* genes was obtained from the Kiwifruit Genome Database. The genomic structures of these genes were displayed as exon/intron organizations by using the Gene Structure Display Server (http://gsds.cbi.pku.edu.cn/, accessed on 1 May 2022) [33].

The online ProtParam tool (https://web.expasy.org/protparam/, accessed on 1 May 2022) was used to analyze the physical and chemical parameters of kiwifruit MDHAR proteins, and the amino acid number, molecular weight, and isoelectric point were computed, respectively. The online Multiple Em for Motif Elicitation tool (MEME tool) (https://meme-suite.org/meme/, accessed on 1 May 2022) was used to discover conserved motifs for the kiwifruit MDHAR proteins using the Zero or One Occurrence Per Sequence method [32].

### 4.5. Analysis of Chromosomal Location and Synteny for MDHAR Genes

The chromosomal locations and genomic directions of the kiwifruit *MDHAR* genes were displayed using the related data obtained from the Kiwifruit Genome Database. TBtools was used to analyze the synteny relationships among *MDHAR* genes within the genome of *A. eriantha* ‘White’. In addition, the synteny relationships for *MDHAR* genes between *A. eriantha* ‘White’ and *A. thaliana* were also analyzed.

### 4.6. Analysis of Relative Expression Levels for Genes in Kiwifruit or Tomato

For frozen samples collected from kiwifruit or tomato plants, they were firstly ground into powder in liquid nitrogen. Then, the powder was used for the extraction of total RNA using a polysaccharides- and polyphenolics-rich RNA isolation kit (Tsingke, Beijing, China). After removing the genomic DNA with Dnase I, the qualified RNA was used to synthesize first-strand cDNA with a M5 Super plus qPCR RT kit (Mei5, Beijing, China). A quantitative real-time PCR (qRT-PCR) analysis was performed for those cDNA samples using a CFX96™ Real-Time PCR machine (C1000 Touch Thermal Cycler; BIO-RAD, Singapore). For kiwifruits and tomatoes, the *AeActin* gene (FG515334.1) and *SlActin* gene (NM_001330119.1) were used as the reference genes, respectively. The relative expression levels of these genes were calculated according the method of Livak and Schmittgen [34]. The primer sequences used for qRT-PCR detection are shown in Table S6 of the Supplementary Files. Three biological repeats were performed for each gene.

### 4.7. Generation of Transgenic Tomato Lines Over-Expressing Kiwifruit AeMDHAR3 Gene

The total RNA of *A. eriantha* ‘Ganmi 6’ was extracted using frozen fruit samples collected from 25th DAFBs. First-strand cDNA was synthesized with the same method as described above. The full CDS of kiwifruit *AeMDHAR3* (genome ID: DTZ79_15g00570) was amplified via the PCR method using the primers of the AeMDHAR3-clone (Table S7 from the Supplementary Files). The DNA segment of the target gene was inserted into pClone-Blunt Simple Vector and was validated via sequencing.

An analysis of sequence similarity between the predicted and the cloned sequences of the *AeMDHAR3* gene was conducted with DNAMAN software (version 6.0, Lynnon Corporation, Vaudreuil, Quebec, Canada). The sequences of AeMDHAR3 and its homologous proteins from other species were used to explore their phylogenetic relationship using the same method as described above. The homologous MDHAR proteins included AcMDHAR (PSS09385.1) from *A. chinensis*, CsMDHAR (KAH9775370.1) from *Citrus sinensis*, MaMDHAR (KAJ4712011.1) from *Melia azedarach*, SlMDHAR (NP_001234013.2) from *S. lycopersicum*, NtMDHAR (NP_001313124.1) from *Nicotiana tabacum*, ArMDHAR (GFZ01457.1) from *A. rufa*, CbMDHAR (PHT57676.1) from *Capsicum baccatum*, DsMDHAR (MCD7455286.1) from *Datura stramonium*, DlMDHAR (XP_052171357.1) from *Diospyros lotus*, GaMDHAR (XP_017650027.1) from *Gossypium arboretum*, GhMDHAR (XP_016739207.2) from *G. hirsutum*, ItMDHAR (XP_031131266.1) from *Ipomoea triloba*, PvMDHAR (XP_031249565.1) from *Pistacia vera*, SdMDHAR (XP_055821606.1) from *S. dulcamara*, SsMDHAR (XP_049376975.1) from *S. stenotomum*, SvMDHAR (XP_049361480.1) from *S. verrucosum*, CcMDHAR (XP_006439479.1) from *C. clementine*, VrMDHAR (XP_034673139.1) from *Vitis riparia*, ZjMDHAR (XP_048320429.1) from *Ziziphus jujuba*, SpMDHAR (XP_015085503.1) from *S. pennellii*, and OeMDHAR (XP_022892959.1) from *Olea europaea*.

The CDS of *AeMDHAR3* was transferred into the plant expression vector, pBWA(V)HS, which contains a CaMV35S promoter. The recombinant pBWA(V)HS-AeMDHAR3 vector was transformed into the *Agrobacterium tumefaciens* GV3101 strain by using the freeze-thaw method. *Agrobacterium tumefaciens*-mediated transformation was performed to infect the cotyledon of the tomato cultivar *S. lycopersicum* ‘Micro-Tom’ according to a previous method [35]. The T_0_ generation transgenic tomato plants were confirmed via a PCR analysis with a *AeMDHAR3*-specific primer, AeMDHAR3-2 (Table S7 in the Supplementary Files).

### 4.8. Analyses of Expression, Enzymatic Activity, and Contents of AsA and DHA in Transgenic Tomato Lines

Three transgenic tomato lines over-expressing the kiwifruit *AeMDHAR3* gene were selected for further analysis. The relative expression levels of *AeMDHAR3* in the leaves of these transgenic lines were analyzed via the qRT-PCR method. For red ripe fruits of over-expressed (OE) (T_1_ generation) and non-transformed wild-type (WT) tomato plants, an activity assay for AsA-accumulation-related enzymes was performed. The activity of MDHAR was detected using a MDHAR Activity Assay Kit (Solarbio, Beijing, China) via spectrophotometry. An activity assay of DHAR was performed according to a previous method [36]. The APX activity was determined according to the previous method of [37]. The AO activity was assayed via a previous method [38]. Additionally, the GR activity was analyzed using a GR Activity Assay Kit (Solarbio). The contents of AsA and DHA were also determined using two Assay Kits (Solarbio). Three biological replicates were performed for the activity assay of each enzyme.

### 4.9. Transcriptome Sequencing and Identification of Differentially Expressed Genes

A RNA-seq method was employed to compare the genome-wide gene expression between the OE−1 transgenic tomato line and the WT plants. For the frozen fruit samples, total RNA was isolated using Trizol reagent kit (Invitrogen, Carlsbad, CA, USA). The qualified eukaryotic mRNA was enriched by Oligo (dT) beads. The enriched mRNA was fragmented using a NEBNext Ultra RNA Library Prep Kit for Illumina (New England Biolabs, Ipswich, MA, USA). The double-stranded cDNA fragments were end-repaired, A base added, and ligated to Illumina sequencing adapters. The purified ligated fragments were subjected to size selection via agarose gel electrophoresis and PCR-amplified. The obtained cDNA library was sequenced using an Illumina Novaseq6000 (Gene Denovo Biotechnology Co., Guangzhou, China).

The transcriptome assembly for clean reads was carried out referring to the tomato genome sequences obtained from the Ensembl Plants database (http://plants.ensembl.org/, accessed on 1 May 2022) [39] between two different groups. The genes with false discovery rates (FDRs) below 0.05 and absolute fold changes ≥2 were considered to be differentially expressed genes (DEGs) between the two groups [40].

### 4.10. Analyses of Gene Ontology Enrichment and KEGG Pathway Enrichment for the Differentially Expressed genes

For the DEGs between the OE−1 line and WT plants, a Gene Ontology (GO) enrichment analysis was performed to find GO terms that were significantly enriched and to filter the DEGs that correspond to specific biological functions. All the DEGs were used to map different GO terms in the Gene Ontology database (http://www.geneontology.org/, accessed on 1 May 2022). The gene number of each GO term was calculated, and the significantly enriched GO terms were identified.

A pathway enrichment analysis based on the KEGG database was conducted to identify significantly enriched metabolic pathways or signal transduction pathways according to the method of Kanehisa and Goto [41], and the significantly enriched KEGG pathways of the DEGs between the two groups were obtained.

### 4.11. Expression Verification for Differentially Expressed Genes via Quantitative Real-Time PCR Analysis

For three significantly enriched KEGG pathways, 12 DEGs (Table 3) were selected for expression verification between OE−1 and WT plants via qRT-PCR analysis; they included 7 genes (*TPS1*, *NUDT14*, *INV1*, *BGLU47*, *DEPE*, *HXK1*, and *AGPS1*) from the pathway of starch and sucrose metabolism (ko00500), 4 genes (*HISN7*, *ALDH2B7*, *ALDH3F1*, and *APX3*) from the pathway of ascorbate and aldarate metabolism (ko00053), and 1 gene (*CYP707A4*) from the pathway of carotenoid biosynthesis (ko00906). The primer sequences used in the qRT-PCR analysis are shown in Table S6 in the Supplementary Files.

### 4.12. Statistical Analysis

The results of most data are indicated as mean ± standard deviation. The significant differences between the OE line(s) and the WT plants were analyzed using the independent-samples T test method at the levels of *p* < 0.05 and *p* < 0.01, respectively, using SPSS software (version 20, SPSS Inc., Chicago, IL, USA). The data of the relative expression levels of *AeMDHAR* genes in different kiwifruit tissues were analyzed via one-way ANOVA and Tukey’s multiple comparison test at a level of *p* < 0.05 using SPSS software.

## 5. Conclusions

Taken together, our present work confirms the critical roles of key genes within the AsA recycling pathway. We found that AsA content in tomato fruit decreased significantly due to the over-expression of the kiwifruit *AeMDHAR3* gene, which is probably achieved by promoting the activities of MDHAR and APX. This study provides direct evidence that the *MDHAR* gene negatively regulates AsA accumulation in fruit, and it identifies a novel feedback regulation scheme for AsA content in kiwifruit. This analysis is useful for future studies on how to effectively improve the nutritional value of kiwifruit and other horticultural crops.

## Figures and Tables

**Table 1:** Genome-wide kiwifruit (*Actinidia eriantha*) *MDHAR* genes and their related features.

Gene Name	Genome ID	Chromosomal Location (Direction)	CDS Length (bp)	Amino Acid Number	Molecular Weight (Da)	Isoelectric Point
*AeMDHAR1*	DTZ79_05g12560	Chr05: 23539803−23546779 (+)	1410	469	50,584.19	8.07
*AeMDHAR2*	DTZ79_12g06930	Chr12: 9554720−9560779 (−)	1395	464	51,413.14	7.09
*AeMDHAR3*	DTZ79_15g00570	Chr15: 590471−598741 (−)	1440	479	51,964.37	8.49
*AeMDHAR4*	DTZ79_20g04830	Chr20: 6405439−6411609 (−)	1506	501	54,574.62	5.69
*AeMDHAR5*	DTZ79_25g06420	Chr25: 15667066−15679146 (−)	1506	501	54,849.56	7.16
*AeMDHAR6*	DTZ79_27g01630	Chr27: 1604069−1610795 (+)	1407	468	50,616.09	7.6
*AeMDHAR7*	DTZ79_27g11730	Chr27: 24128673−24134913 (−)	1383	460	50,130.73	8.3

**Table 2:** The read qualities of fruit samples obtained from RNA-seq analysis.

Sample	Raw Reads	Clean Reads	Total Bases (Gb)	Filtered Bases (Gb)	Q20 (Percentage of Base with Qphred Value ≥ 20)	Q30 (Percentage of Base with Qphred Value ≥ p30)	GC (Percentage of GC Content)
WT−1	46,335,034	46,121,526	6.47	6.42	97.01	91.75	42.23
WT−2	50,712,724	50,513,766	7.08	7.03	97.8	93.41	42.21
WT−3	91,821,512	91,422,210	12.83	12.73	96.86	91.44	42.22
OE−1−1	49,241,644	49,011,976	6.88	6.82	96.94	91.62	42.3
OE−1−2	46,022,396	45,816,832	6.43	6.37	96.94	91.61	42.17
OE−1−3	41,615,986	41,434,366	5.81	5.76	97.07	91.87	42.1

**Table 3:** The information of selected differentially expressed genes in transgenic tomato plants and their KEGG pathways.

Genome ID	Gene Name	Symbol	Description	KEGG Pathway
Solyc07g062140.3	*SlTPS1*	TPS1	Trehalose-phosphate synthase 1	ko00500/starch and sucrose metabolism
Solyc08g079820.3	*SlNUDT14*	NUDT14	Predicted: nudix hydrolase 14, chloroplastic	ko00500/starch and sucrose metabolism
Solyc09g010080.3	*SlINV1*	INV1	Beta-fructofuranosidase	ko00500/starch and sucrose metabolism
Solyc02g080300.3	*SlBGLU47*	BGLU47	Predicted: beta-glucosidase 18	ko00500/starch and sucrose metabolism
Solyc04g053120.3	*SlDPEP*	DPEP	Predicted: 4-alpha-glucanotransferase, chloroplastic/amyloplastic	ko00500/starch and sucrose metabolism
Solyc03g121070.3	*SlHXK1*	HXK1	Hexokinase	ko00500/starch and sucrose metabolism
Solyc07g056140.3	*SlAGPS1*	AGPS1	Glucose-1-phosphate adenylyltransferase small subunit, chloroplastic	ko00500/starch and sucrose metabolism
Solyc04g078900.3	*SlCYP707A4*	CYP707A4	ABA 8’-hydroxylase	ko00906/carotenoid biosynthesis
Solyc01g109930.4	*SlHISN7*	HISN7	Predicted: bifunctional phosphatase IMPL2, chloroplastic	ko00053/ascorbate and aldarate metabolism
Solyc05g005700.4	*SlALDH2B7*	ALDH2B7	Aldehyde dehydrogenase family 2 member B7d	ko00053/ascorbate and aldarate metabolism
Solyc02g084640.4	*SlALDH3F1*	ALDH3F1	Predicted: aldehyde dehydrogenase family 3 member F1	ko00053/ascorbate and aldarate metabolism
Solyc01g111510.3	*SlAPX3*	APX3	L-ascorbate peroxidase 3, peroxisomal	ko00053/ascorbate and aldarate metabolism

## Data Availability

Data are available from the corresponding authors upon reasonable request.

## Supplementary Materials

The supporting information can be downloaded at: https://www.mdpi.com/article/10.3390/ijms242417182/s1.
