# Supplementary material for: Kiwifruit Monodehydroascorbate Reductase 3 Gene Negatively Regulates the Accumulation of Ascorbic Acid in Fruit of Transgenic Tomato Plants
Source: Int J Mol Sci. 2023 Dec 6;24(24):17182. doi: 10.3390/ijms242417182 (PMC10742914; doi:10.3390/ijms242417182)
Supplement: Supplementary file 1 [file ijms-24-17182-s001.zip › Table S1.docx]

**Table S1.** The information of the conserved domains identified in kiwifruit (*Actinidia eriantha*) MDHAR proteins.

| Protein | Domain | Accession number | Description | Interval | E-value |
| --- | --- | --- | --- | --- | --- |
| AeMDHAR1 | Pyr_Redox_2 | Pfam07992 | Pyridine nucleotide-disulphide oxidoreductase | 29-309 | 4.80e-44 |
| AeMDHAR2 | Pyr_Redox_2 | Pfam07992 | Pyridine nucleotide-disulphide oxidoreductase | 6-352 | 1.01e-34 |
| AeMDHAR3 | Pyr_Redox_2 | Pfam07992 | Pyridine nucleotide-disulphide oxidoreductase | 79-370 | 7.00e-44 |
| AeMDHAR4 | Pyr_Redox_2 | Pfam07992 | Pyridine nucleotide-disulphide oxidoreductase | 62-328 | 1.54e-51 |
| AeMDHAR5 | Pyr_Redox_2 | Pfam07992 | Pyridine nucleotide-disulphide oxidoreductase | 71-375 | 6.82e-44 |
| AeMDHAR6 | Pyr_Redox_2 | Pfam07992 | Pyridine nucleotide-disulphide oxidoreductase | 29-308 | 5.80e-33 |
| AeMDHAR7 | Pyr_Redox_2 | Pfam07992 | Pyridine nucleotide-disulphide oxidoreductase | 29-298 | 6.29e-28 |
