# Supplementary material for: Kiwifruit Monodehydroascorbate Reductase 3 Gene Negatively Regulates the Accumulation of Ascorbic Acid in Fruit of Transgenic Tomato Plants
Source: Int J Mol Sci. 2023 Dec 6;24(24):17182. doi: 10.3390/ijms242417182 (PMC10742914; doi:10.3390/ijms242417182)
Supplement: Supplementary file 1 [file ijms-24-17182-s001.zip › Table S3.docx]

**Table S3.** The information of motifs identified in kiwifruit (*Actinidia eriantha*) MDHAR proteins.

| Motif name | Length (aa) | Motif consensus |
| --- | --- | --- |
| Motif 1 | 50 | KGGIKVDGRFRTSVPSVYAVGDVAAFPVKLYGESRRVEHVDHARKSAKHA |
| Motif 2 | 50 | YVIIGGGVAAGYAAREFVKRGVSQGELCIISKESVAPYERPALSKAYLFP |
| Motif 3 | 29 | APARLPGFHTCVGSGGERLTPEWYKEKGI |
| Motif 4 | 30 | VINKJDVTMVFPEAHCMPRLFTPKIAAYYE |
| Motif 5 | 32 | PFFYSRVFTLSWQFYGDNVGEIVHFGDFSPTT |
| Motif 6 | 40 | DGKVTAVKLRDGRVLEADMVVVGIGIRPNTALFEGQVTLD |
| Motif 7 | 50 | FGAYWISKGHLVGSFLEGGTKEQYEAJSKVTRLKPAIEDLAELQRQGLGF |
| Motif 8 | 25 | NVFYLRDVADADRLVEVMQSCTKGK |
| Motif 9 | 29 | KTLLTAAGELLKYKILIIATGATALRLED |
| Motif 10 | 41 | VGGPALALGRPLYAWHATAGVILAASVAVFAYWYGRKRRRW |
