# Supplementary material for: Kiwifruit Monodehydroascorbate Reductase 3 Gene Negatively Regulates the Accumulation of Ascorbic Acid in Fruit of Transgenic Tomato Plants
Source: Int J Mol Sci. 2023 Dec 6;24(24):17182. doi: 10.3390/ijms242417182 (PMC10742914; doi:10.3390/ijms242417182)
Supplement: Supplementary file 1 [file ijms-24-17182-s001.zip › Table S4.docx]

**Table S4.** Coding sequence and the encoded protein sequence of *AeMDHAR3* gene cloned form *Actinidia eriantha* ‘Ganmi 6’.

| Gene | Gene sequence (CDS) | Protein sequence |
| --- | --- | --- |
| *AeMDHAR3* | ATGTCTTCAGTTTGTAAATTAATGGCGAGCATGTCGAACTCGTTGTCTCTAAAGCACGGTCTGTCTCTCTGGTGCCCTCAGTCCGCTTCTGTTAATCGAATTCGCCACCAGATTCCTTCTAGAAGCTTCCGCCGAAGCTTCGCAGCGGCTTCTTACGCCAATGAGAATCGCGAGTTTGTGATTGTTGGGGGTGGAAATGCAGCTGGATATGCAGCTCGTACTTTCGTTGAGCATGGGATGGCCAATGGAAAGCTGTGTATTGTAACAAAAGAGGAACATGCACCTTATGAGAGGCCAGCTTTGACGAAAGCTTACTTGTTCCCTACAGATAAAAAGCCTGCTCGCTTACCTGGCTTTCATACATGTGTTGGATCGGGTGGCGAAAGGCAGACCCCTGAGTGGTATAACGAACAGGGCATTGAGATGTTGTACAAGGATCCTGTTACAGGTATTGATGTCGAAAAGCAAACTCTGACAACAAATTCAGGAAAATTACTAAAGTATGGGTCTCTTATTGTTGCCACCGGATGTACAGCTTCAAGATTTCCAGACAAGATTGGTGGAAACTTACCTGGTGTTCACTATATTCGAGATGTTGCAGATGCTGATTTGCTGATCTCATCACTGGAGAAAGCACAGAAGGTTGTCGTGGTTGGTGGTGGTTACATTGGCATGGAGGTTGCTGCTGCAGCTGTTGGCTGGAAACTTGATACGACGATCATATTTCCAGAGAATCATCTTCTGCAAAGATTGTTTACTCCTTCCCTCGCCGAGAAATATGAAAGACTTTACCAGGAGAATGGCGTCAAATTCTTGAAGGGTGCCTCCATAAAGAACTTAGAAGCTGGTCCTGACGGATGTGTGGCTGCTGTTAGACTTGAGAATGGGTCTACAATAGAAGCAGACACAATTGTTATTGGTATTGGAGCTAGACCTGCTGTCAGTCCTTTTGAAGCGATGGTGGGGTTGAACAAGCAAGTTGGTGGAATACAGGTTGATGGTCAGTTCCGAACAAGTGTCCCTGGAATTTTTGCAATTGGAGATGTAGCGGCATTCCCCTTAAAGATGTATGATCGCATTGCAAGAGTCGAACATGTTGATCATGCTCGCCGATCTGCCCAGCATTGCGTTAATGCACTACTGAGTGCAAAAACTCACACGTACGATTATCTACCATATTTCTACTCGAGGGTCTTTGAGTATGAAGGAAGCCCGAGGAAAGTTTGGTGGCAGTTTTTTGGGGACAATGTTGGCGAGATTGTCGAAATTGGAAATTTTGACCCTAAGATTGCTACTTTCTGGATAGACTCTGGTAAGCTGAAAGGAATTCTTCTTGAAAGTGGAAGTCCTGAGGAATTTCAACTTCTTCCTAAGCTTGCAAGGAGCCAGCCTAATGTTGATAAAGCCAAACTCCAGAGTGCATCATCGGTTGAGGCGGCACTACAAATTGCTCAATCTTCATTGTAA | MSSVCKLMASMSNSLSLKHGLSLWCPQSASVNRIRHQIPSRSFRRSFAAASYANENREFVIVGGGNAAGYAARTFVEHGMANGKLCIVTKEEHAPYERPALTKAYLFPTDKKPARLPGFHTCVGSGGERQTPEWYNEQGIEMLYKDPVTGIDVEKQTLTTNSGKLLKYGSLIVATGCTASRFPDKIGGNLPGVHYIRDVADADLLISSLEKAQKVVVVGGGYIGMEVAAAAVGWKLDTTIIFPENHLLQRLFTPSLAEKYERLYQENGVKFLKGASIKNLEAGPDGCVAAVRLENGSTIEADTIVIGIGARPAVSPFEAMVGLNKQVGGIQVDGQFRTSVPGIFAIGDVAAFPLKMYDRIARVEHVDHARRSAQHCVNALLSAKTHTYDYLPYFYSRVFEYEGSPRKVWWQFFGDNVGEIVEIGNFDPKIATFWIDSGKLKGILLESGSPEEFQLLPKLARSQPNVDKAKLQSASSVEAALQIAQSSL |
