# Supplementary material for: Kiwifruit Monodehydroascorbate Reductase 3 Gene Negatively Regulates the Accumulation of Ascorbic Acid in Fruit of Transgenic Tomato Plants
Source: Int J Mol Sci. 2023 Dec 6;24(24):17182. doi: 10.3390/ijms242417182 (PMC10742914; doi:10.3390/ijms242417182)
Supplement: Supplementary file 1 [file ijms-24-17182-s001.zip › Table S5.docx]

**Table S5.** The information of significantly enriched KEGG pathways for the differentially expressed genes between OE-1 line and the wild-type tomato plants.

| KEGG pathway | Gene number | class | ID | Pvalue | Qvalue | Up | Down |
| --- | --- | --- | --- | --- | --- | --- | --- |
| Biosynthesis of secondary metabolites | 120 | Metabolism | ko01110 | 3.06E-05 | 0.003249 | 92 | 28 |
| Cysteine and methionine metabolism | 18 | Metabolism | ko00270 | 9.53E-05 | 0.003531 | 11 | 7 |
| Starch and sucrose metabolism | 21 | Metabolism | ko00500 | 9.99E-05 | 0.003531 | 19 | 2 |
| Metabolic pathways | 199 | Metabolism | ko01100 | 0.000511 | 0.013542 | 157 | 42 |
| Glycerolipid metabolism | 13 | Metabolism | ko00561 | 0.0012 | 0.025432 | 12 | 1 |
| Plant-pathogen interaction | 38 | Organismal Systems | ko04626 | 0.002235 | 0.039478 | 35 | 3 |
| Linoleic acid metabolism | 6 | Metabolism | ko00591 | 0.002793 | 0.042296 | 4 | 2 |
| Amino sugar and nucleotide sugar metabolism | 22 | Metabolism | ko00520 | 0.003846 | 0.049428 | 13 | 9 |
| Valine, leucine and isoleucine degradation | 11 | Metabolism | ko00280 | 0.004197 | 0.049428 | 10 | 1 |
| Arginine and proline metabolism | 10 | Metabolism | ko00330 | 0.005294 | 0.0548 | 8 | 2 |
| Brassinosteroid biosynthesis | 5 | Metabolism | ko00905 | 0.005687 | 0.0548 | 5 | 0 |
| beta-Alanine metabolism | 10 | Metabolism | ko00410 | 0.006441 | 0.056898 | 9 | 1 |
| Carbon fixation in photosynthetic organisms | 11 | Metabolism | ko00710 | 0.010918 | 0.089027 | 7 | 4 |
| Histidine metabolism | 5 | Metabolism | ko00340 | 0.013945 | 0.105584 | 4 | 1 |
| Fatty acid degradation | 9 | Metabolism | ko00071 | 0.019922 | 0.14078 | 8 | 1 |
| Carotenoid biosynthesis | 6 | Metabolism | ko00906 | 0.024941 | 0.165235 | 5 | 1 |
| Ascorbate and aldarate metabolism | 8 | Metabolism | ko00053 | 0.028145 | 0.175491 | 7 | 1 |
| Inositol phosphate metabolism | 10 | Metabolism | ko00562 | 0.031134 | 0.183344 | 9 | 1 |
| Pyruvate metabolism | 11 | Metabolism | ko00620 | 0.040787 | 0.225038 | 9 | 2 |
| Carbon metabolism | 25 | Metabolism | ko01200 | 0.04246 | 0.225038 | 20 | 5 |
| Other glycan degradation | 3 | Metabolism | ko00511 | 0.044724 | 0.225749 | 3 | 0 |
| Pentose and glucuronate interconversions | 13 | Metabolism | ko00040 | 0.047283 | 0.227818 | 10 | 3 |
| Ubiquinone and other terpenoid-quinone biosynthesis | 6 | Metabolism | ko00130 | 0.052707 | 0.228505 | 5 | 1 |
| Tropane, piperidine and pyridine alkaloid biosynthesis | 5 | Metabolism | ko00960 | 0.053394 | 0.228505 | 4 | 1 |
| Plant hormone signal transduction | 30 | Environmental Information Processing | ko04075 | 0.053893 | 0.228505 | 24 | 6 |
| Cyanoamino acid metabolism | 6 | Metabolism | ko00460 | 0.057122 | 0.232882 | 6 | 0 |
| Butanoate metabolism | 4 | Metabolism | ko00650 | 0.06303 | 0.247452 | 4 | 0 |
| Phenylpropanoid biosynthesis | 22 | Metabolism | ko00940 | 0.06656 | 0.25 | 17 | 5 |
| Steroid biosynthesis | 5 | Metabolism | ko00100 | 0.069957 | 0.25 | 4 | 1 |
| Tyrosine metabolism | 7 | Metabolism | ko00350 | 0.070755 | 0.25 | 6 | 1 |
| MAPK signaling pathway - plant | 20 | Environmental Information Processing | ko04016 | 0.073474 | 0.251234 | 13 | 7 |
| Sphingolipid metabolism | 4 | Metabolism | ko00600 | 0.077583 | 0.2524 | 4 | 0 |
| Riboflavin metabolism | 3 | Metabolism | ko00740 | 0.081248 | 0.2524 | 2 | 1 |
| Glycosphingolipid biosynthesis - globo and isoglobo series | 2 | Metabolism | ko00603 | 0.0826 | 0.2524 | 2 | 0 |
| Phenylalanine metabolism | 10 | Metabolism | ko00360 | 0.08334 | 0.2524 | 6 | 4 |
| Glycerophospholipid metabolism | 10 | Metabolism | ko00564 | 0.087248 | 0.256898 | 10 | 0 |
| Glycosaminoglycan degradation | 3 | Metabolism | ko00531 | 0.091892 | 0.262353 | 3 | 0 |
| Tryptophan metabolism | 6 | Metabolism | ko00380 | 0.094051 | 0.262353 | 5 | 1 |
| Synthesis and degradation of ketone bodies | 2 | Metabolism | ko00072 | 0.099643 | 0.268258 | 2 | 0 |
| Isoquinoline alkaloid biosynthesis | 4 | Metabolism | ko00950 | 0.102241 | 0.268258 | 3 | 1 |
| Biosynthesis of amino acids | 19 | Metabolism | ko01230 | 0.10376 | 0.268258 | 14 | 5 |
| Galactose metabolism | 6 | Metabolism | ko00052 | 0.126686 | 0.319732 | 6 | 0 |
| Sulfur metabolism | 4 | Metabolism | ko00920 | 0.139906 | 0.344885 | 3 | 1 |
| Cutin, suberine and wax biosynthesis | 4 | Metabolism | ko00073 | 0.150084 | 0.361566 | 2 | 2 |
| Taurine and hypotaurine metabolism | 2 | Metabolism | ko00430 | 0.155405 | 0.366066 | 2 | 0 |
| Fructose and mannose metabolism | 7 | Metabolism | ko00051 | 0.164833 | 0.370448 | 6 | 1 |
| Alanine, aspartate and glutamate metabolism | 5 | Metabolism | ko00250 | 0.169113 | 0.370448 | 4 | 1 |
| Phosphatidylinositol signaling system | 8 | Environmental Information Processing | ko04070 | 0.170326 | 0.370448 | 8 | 0 |
| Arginine biosynthesis | 4 | Metabolism | ko00220 | 0.171245 | 0.370448 | 2 | 2 |
| Phenylalanine, tyrosine and tryptophan biosynthesis | 5 | Metabolism | ko00400 | 0.17833 | 0.37806 | 3 | 2 |
| Biosynthesis of unsaturated fatty acids | 4 | Metabolism | ko01040 | 0.20475 | 0.425559 | 3 | 1 |
| ABC transporters | 4 | Environmental Information Processing | ko02010 | 0.216322 | 0.43638 | 4 | 0 |
| Fatty acid metabolism | 8 | Metabolism | ko01212 | 0.219314 | 0.43638 | 7 | 1 |
| Nicotinate and nicotinamide metabolism | 3 | Metabolism | ko00760 | 0.222307 | 0.43638 | 3 | 0 |
| Glycine, serine and threonine metabolism | 6 | Metabolism | ko00260 | 0.232027 | 0.447179 | 6 | 0 |
| Glycolysis / Gluconeogenesis | 11 | Metabolism | ko00010 | 0.241771 | 0.454194 | 9 | 2 |
| Caffeine metabolism | 1 | Metabolism | ko00232 | 0.244237 | 0.454194 | 1 | 0 |
| Lysine degradation | 5 | Metabolism | ko00310 | 0.268384 | 0.490496 | 4 | 1 |
| Ether lipid metabolism | 3 | Metabolism | ko00565 | 0.281809 | 0.506301 | 3 | 0 |
| Nitrogen metabolism | 3 | Metabolism | ko00910 | 0.312113 | 0.538121 | 3 | 0 |
| Diterpenoid biosynthesis | 4 | Metabolism | ko00904 | 0.313744 | 0.538121 | 1 | 3 |
| Thiamine metabolism | 2 | Metabolism | ko00730 | 0.318299 | 0.538121 | 2 | 0 |
| Phagosome | 7 | Cellular Processes | ko04145 | 0.319827 | 0.538121 | 6 | 1 |
| Terpenoid backbone biosynthesis | 4 | Metabolism | ko00900 | 0.37663 | 0.623249 | 2 | 2 |
| Glyoxylate and dicarboxylate metabolism | 6 | Metabolism | ko00630 | 0.385644 | 0.623249 | 5 | 1 |
| Glutathione metabolism | 8 | Metabolism | ko00480 | 0.388061 | 0.623249 | 8 | 0 |
| Peroxisome | 7 | Cellular Processes | ko04146 | 0.409971 | 0.64861 | 6 | 1 |
| Pentose phosphate pathway | 4 | Metabolism | ko00030 | 0.451258 | 0.699853 | 4 | 0 |
| Folate biosynthesis | 2 | Metabolism | ko00790 | 0.455565 | 0.699853 | 2 | 0 |
| Sulfur relay system | 1 | Genetic Information Processing | ko04122 | 0.489521 | 0.731209 | 1 | 0 |
| Fatty acid elongation | 3 | Metabolism | ko00062 | 0.489772 | 0.731209 | 3 | 0 |
| Protein processing in endoplasmic reticulum | 14 | Genetic Information Processing | ko04141 | 0.598694 | 0.88141 | 12 | 2 |
| Sesquiterpenoid and triterpenoid biosynthesis | 2 | Metabolism | ko00909 | 0.635161 | 0.909117 | 2 | 0 |
| Selenocompound metabolism | 1 | Metabolism | ko00450 | 0.635434 | 0.909117 | 1 | 0 |
| One carbon pool by folate | 1 | Metabolism | ko00670 | 0.655336 | 0.909117 | 1 | 0 |
| Indole alkaloid biosynthesis | 1 | Metabolism | ko00901 | 0.655336 | 0.909117 | 0 | 1 |
| Arachidonic acid metabolism | 1 | Metabolism | ko00590 | 0.674153 | 0.909117 | 0 | 1 |
| Citrate cycle (TCA cycle) | 3 | Metabolism | ko00020 | 0.674908 | 0.909117 | 3 | 0 |
| Propanoate metabolism | 3 | Metabolism | ko00640 | 0.685308 | 0.909117 | 3 | 0 |
| Flavonoid biosynthesis | 4 | Metabolism | ko00941 | 0.687816 | 0.909117 | 3 | 1 |
| alpha-Linolenic acid metabolism | 3 | Metabolism | ko00592 | 0.695457 | 0.909117 | 1 | 2 |
| Fatty acid biosynthesis | 3 | Metabolism | ko00061 | 0.705358 | 0.909117 | 2 | 1 |
| Base excision repair | 2 | Genetic Information Processing | ko03410 | 0.711856 | 0.909117 | 2 | 0 |
| Monoterpenoid biosynthesis | 1 | Metabolism | ko00902 | 0.724677 | 0.914473 | 0 | 1 |
| Circadian rhythm - plant | 2 | Organismal Systems | ko04712 | 0.774598 | 0.95473 | 1 | 1 |
| Valine, leucine and isoleucine biosynthesis | 1 | Metabolism | ko00290 | 0.780096 | 0.95473 | 1 | 0 |
| Stilbenoid, diarylheptanoid and gingerol biosynthesis | 3 | Metabolism | ko00945 | 0.783599 | 0.95473 | 2 | 1 |
| Porphyrin and chlorophyll metabolism | 2 | Metabolism | ko00860 | 0.817443 | 0.984648 | 2 | 0 |
| 2-Oxocarboxylic acid metabolism | 2 | Metabolism | ko01210 | 0.876563 | 0.999996 | 1 | 1 |
| Ubiquitin mediated proteolysis | 7 | Genetic Information Processing | ko04120 | 0.87785 | 0.999996 | 5 | 2 |
| DNA replication | 3 | Genetic Information Processing | ko03030 | 0.905099 | 0.999996 | 0 | 3 |
| SNARE interactions in vesicular transport | 1 | Genetic Information Processing | ko04130 | 0.932571 | 0.999996 | 1 | 0 |
| Endocytosis | 8 | Cellular Processes | ko04144 | 0.942775 | 0.999996 | 8 | 0 |
| Photosynthesis | 7 | Metabolism | ko00195 | 0.943402 | 0.999996 | 5 | 2 |
| Pyrimidine metabolism | 7 | Metabolism | ko00240 | 0.946831 | 0.999996 | 5 | 2 |
| Zeatin biosynthesis | 4 | Metabolism | ko00908 | 0.949704 | 0.999996 | 4 | 0 |
| Proteasome | 1 | Genetic Information Processing | ko03050 | 0.963751 | 0.999996 | 1 | 0 |
| Mismatch repair | 2 | Genetic Information Processing | ko03430 | 0.985023 | 0.999996 | 2 | 0 |
| Ribosome biogenesis in eukaryotes | 1 | Genetic Information Processing | ko03008 | 0.994075 | 0.999996 | 1 | 0 |
| RNA degradation | 2 | Genetic Information Processing | ko03018 | 0.996466 | 0.999996 | 2 | 0 |
| Purine metabolism | 5 | Metabolism | ko00230 | 0.997261 | 0.999996 | 4 | 1 |
| Spliceosome | 4 | Genetic Information Processing | ko03040 | 0.997869 | 0.999996 | 4 | 0 |
| RNA polymerase | 1 | Genetic Information Processing | ko03020 | 0.998203 | 0.999996 | 1 | 0 |
| RNA transport | 3 | Genetic Information Processing | ko03013 | 0.999127 | 0.999996 | 2 | 1 |
| mRNA surveillance pathway | 1 | Genetic Information Processing | ko03015 | 0.999615 | 0.999996 | 1 | 0 |
| Oxidative phosphorylation | 5 | Metabolism | ko00190 | 0.999996 | 0.999996 | 5 | 0 |
