# Supplementary material for: Kiwifruit Monodehydroascorbate Reductase 3 Gene Negatively Regulates the Accumulation of Ascorbic Acid in Fruit of Transgenic Tomato Plants
Source: Int J Mol Sci. 2023 Dec 6;24(24):17182. doi: 10.3390/ijms242417182 (PMC10742914; doi:10.3390/ijms242417182)
Supplement: Supplementary file 1 [file ijms-24-17182-s001.zip › Table S6.docx]

**Table S6.** Primer sequences of related genes used for quantitative real-time PCR analysis.

| Gene | Gene ID | Forward sequence (5'-3') | Reverse sequence (5'-3') |
| --- | --- | --- | --- |
| *AeMDHAR1* | DTZ79_05g12560 | GGTGCCAATGAGGAAAGGTT | CAATAACAACAGCATTCCCACC |
| *AeMDHAR2* | DTZ79_12g06930 | GTGGGTGATGTTGCTACTTTCC | TCCGATGATGGGTTGTTGTCT |
| *AeMDHAR3* | DTZ79_15g00570 | TCACTGGAGAAAGCACAGAAGG | GCGAGGGAAGGAGTAAACAATC |
| *AeMDHAR4* | DTZ79_20g04830 | AATGGAAAGGCTGTGATTGTTG | CAGCCACAGTTCCCTTGATAAT |
| *AeMDHAR5* | DTZ79_25g06420 | ATTGTTTACTCCTTCCCTTGCC | GACCATCAACCTGTATTCCACC |
| *AeMDHAR6* | DTZ79_27g01630 | TCAAAGTGAACGGGCGAATG | TGGAATAGAAGAACGGCAGGTA |
| *AeMDHAR7* | DTZ79_27g11730 | ATCTAAGGGACGGGAATCATCT | GGCAACATCACCAACAGCATA |
| *AeActin* | FG515334.1 | GTGCTCAGTGGTGGTTCAA | GACGCTGTATTTCCTCTCAG |
| *TPS1* | Solyc07g062140.3 | CGAAGAAGGTGATGTGGTATGG | TCCGAGGATGGAAAGGGTG |
| *NUDT14* | Solyc08g079820.3 | CATCAATCTCCCCACCCAA | CCGTGCGAAGACAATACCAG |
| *INV1* | Solyc09g010080.3 | GGGTTATTTGGGGTTGGTCA | CCTTTTGCTTCCTTAGGGTTTC |
| *BGLU47* | Solyc02g080300.3 | ATTCTGCCCAAAGGGACATT | GGTTAGGCTCATTGATGGTTGT |
| *DPEP* | Solyc04g053120.3 | AAAACCCTCATTTGCCTCACA | CCCAAGCCCAAGAACATCC |
| *HXK1* | Solyc03g121070.3 | ATTTGTTGCTGCGGAAGAGG | AACATCTTGACCAACCGCATC |
| *AGPS1* | Solyc07g056140.3 | AATCGCCACCTTTCACGG | ATGCTCCTCAAACAACCACAGA |
| *CYP707A4* | Solyc04g078900.3 | AAAAGGATGGAAGGTGATGCC | AGGAAAGAACTCTGGGTTGTGA |
| *HISN7* | Solyc01g109930.4 | GAGATTGCCTTTGCTCGTGTT | GCCTTTCCAGTCGGTTATTGT |
| *ALDH2B7* | Solyc05g005700.4 | GGCGGCTCGTGTATTTCTC | TCCACATTGACAGTAGGCTTGA |
| *ALDH3F1* | Solyc02g084640.4 | AATCCTCCCCTTGATGCTGA | GCCTTGAGTTTATGAACGGAAT |
| *APX3* | Solyc01g111510.3 | ACTCACGGTGCTAATAATGGCT | CCTTTGGAGAAACACTGGAATC |
| *SlActin* | NM_001330119.1 | CTGGATTGGAGGCTCTATC | GCATCTCTGGTCCAGTAGG |
