# Supplementary material for: Kiwifruit Monodehydroascorbate Reductase 3 Gene Negatively Regulates the Accumulation of Ascorbic Acid in Fruit of Transgenic Tomato Plants
Source: Int J Mol Sci. 2023 Dec 6;24(24):17182. doi: 10.3390/ijms242417182 (PMC10742914; doi:10.3390/ijms242417182)
Supplement: Supplementary file 1 [file ijms-24-17182-s001.zip › Table S7.docx]

**Table S7.** Primer sequences used for gene clone and the construction of plant expression vector.

| Primer name | Forward sequence (5'-3') | Reverse sequence (5'-3') |
| --- | --- | --- |
| AeMDHAR3-clone | ATGTCTTCAGTTGGTAAATTAAT | TTACAATGAAGATTGAGCAATT |
| AeMDHAR3-2 | CAGTCGTCTCACAACATGTCTTCAGTTTGTAAATT | CAGTCGTCTCATACATTACAATGAAGATTGAGCAA |
