# Supplementary figures and images for: Kiwifruit Monodehydroascorbate Reductase 3 Gene Negatively Regulates the Accumulation of Ascorbic Acid in Fruit of Transgenic Tomato Plants
Source: Int J Mol Sci. 2023 Dec 6;24(24):17182. doi: 10.3390/ijms242417182 (PMC10742914; doi:10.3390/ijms242417182)

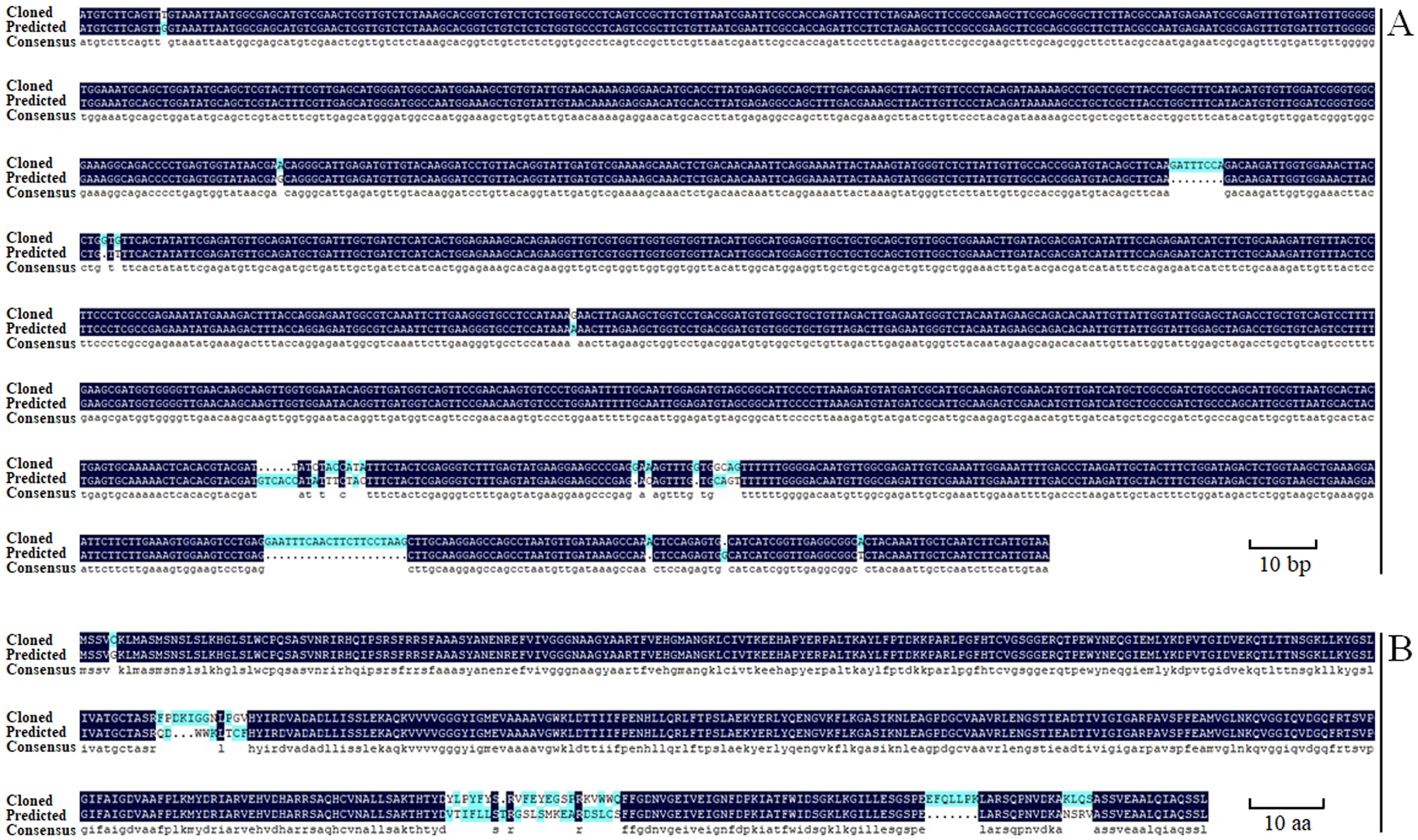

Supplement: Supplementary file 1 [file ijms-24-17182-s001.zip › Figure S1.tif]
